# First nearly complete skull of *Gallotia auaritae* (lower-middle Pleistocene, Squamata, Gallotiinae) and a morphological phylogenetic analysis of the genus *Gallotia*

**DOI:** 10.1038/s41598-019-52244-z

**Published:** 2019-11-12

**Authors:** Penélope Cruzado-Caballero, Carolina Castillo Ruiz, Arnau Bolet, Juan Ramón Colmenero, Julio De la Nuez, Ramón Casillas, Sergio Llacer, Federico Bernardini, Josep Fortuny

**Affiliations:** 1 CONICET, Universidad Nacional de Río Negro. Instituto de Investigación en Paleobiología y Geología, General Roca. 8332, Rio Negro, Argentina; 2 Departamento de Biología Animal, Edafología y Geología, Universidad de La Laguna, Av. Astrofisico Francisco Sánchez, 2, 38206, San Cristóbal de La Laguna, Santa Cruz de Tenerife, Spain; 3 Institut Català de Paleontologia Miquel Crusafont (ICP), Universitat Autònoma de Barcelona (UAB), Edifici ICTA-ICP, Carrer de les Columnes s/n, Campus de la UAB, 08193 Cerdanyola del Vallès, Barcelona, Spain; 4 School of Earth Sciences, University of Bristol, Life Sciences Building, 24 Tyndall Avenue, Bristol, BS8 1TQ UK; 5 Departamento de Geología, Facultad de Ciencias, Universidad de Salamanca, Plaza de la Merced s/n, 37008 Salamanca, Spain; 6 Centro Fermi, Museo Storico della Fisica e Centro di Studi e Ricerche “Enrico Fermi”, Piazza del Viminale 1, 00184 Roma, Italy; 7 Multidisciplinary Laboratory, The “Abdus Salam” International Centre for Theoretical Physics, Strada Costiera 11, 34151 Trieste, Italy

**Keywords:** Palaeontology, Evolution, Ecology, Evolutionary ecology

## Abstract

The Canary Islands are an Atlantic archipelago known for its high number of endemic species. Among the most known endemic vertebrate species are the giant lizards of the genus *Gallotia*. We describe the cranial osteology of the first almost complete and articulated fossil skull of the taxon *Gallotia auaritae*, recovered from the lower-middle Pleistocene of the La Palma island. In this work, X-ray computed microtomography images were used to perform an exhaustive phylogenetic analysis where most of the extant and fossil species of the genus *Gallotia* were included for first time. This analysis recovered a monophyletic *Gallotia* clade with similar topology to that of molecular analyses. The newly described specimen shares some characters with the group formed by *G. bravoana, G. intermedia* and *G. simonyi*, *G. auaritae*, and its position is compatible with a referral to the latter. Our study adds new important data to the poorly known cranial morphology of *G*. *auaritae*, and the phylogenetic analysis reveals an unexpected power of resolution to obtain a morphology-based phylogeny for the genus *Gallotia*, for inferring the phylogenetic position of extinct species and for helping in the identification of fossil specimens.

## Introduction

The Canary Islands are a volcanic archipelago located at the northwest of Africa and formed along the Neogene and Quaternary periods. The origin of this archipelago is related to a hot spot^1^, where the islands appeared in a phased manner. Fuerteventura was the first island to emerge (20 Ma, Miocene)^2^ whereas El Hierro island was the last (1.1 Ma, Pleistocene)^1^. This archipelago is one of the greatest natural labs for the study of volcanology on our planet. This special geological setting has favoured the presence of a high number of endemic species of plants, invertebrates and vertebrates in the archipelago. For instance, the squamate lizard genus *Gallotia* forms an emblematic and endemic clade with multiple extinct and extant described species ranging from mid-sized to giant forms. Regarding the islands fossil record, the oldest vertebrate fossils are a snake vertebra (Boidae indet.) and large bird remains (bones and eggshells) belonging to the Miocene (23–5 Ma) of Lanzarote and tortoise remains (bones and eggshells) from the Miocene (23–5 Ma) of Fuerteventura and Pliocene (5–2.58 Ma) of Lanzarote and Fuerteventura^3^.

It is beyond the scope of this paper to exhaustively review the convoluted taxonomical history of the giant forms of the genus *Gallotia*. However, in order to be consistent along the paper, a decision on the identity of the form (or forms) in each island needs to be taken. We briefly expose here our decisions and the reasons that lead us in this direction. So far there is no disagreement in that the giant form represented in Gran Canaria is *Gallotia stehlini*^4^. Lanzarote and Fuerteventura lack giant forms, except for the introduced *G*. *stehlini* in the latter. The situation is, however, more complicated in the rest of islands, where multiple subspecies have been described and occasionally used at the rank of species. The study of Barahona *et al*.^5^ shows the limitations of the diagnostic characters used to identify some of the previously recognised taxa. They regarded *Gallotia goliath*^6^, including *Gallotia maxima* which they considered indiferentiable from the former, as a synonym of *Gallotia simonyi*^7^. According to Barahona *et al*.^5^ and Mateo *et al*.^8^, the characters formerly used in the diagnosis of extinct species and subspecies are either related to a large size or fall within the range of intraspecific variation of the extant *G*. *simonyi*. Despite this, soon after this publication some of the same authors published a work^8^ where four subspecies of *G*. *simonyi* were recognised: *G*. *simonyi simonyi* in El Hierro; *G*. *simonyi goliath* in Tenerife; *G*. *simonyi bravoana* in La Gomera; and their newly described subspecies *G*. *simonyi auaritae* in La Palma. Some of these subspecies have been subsequently given the rank of species: *G*. *simonyi goliath* recovered its species status (*G*. *goliath*) after the molecular analysis^9^ of the mummified specimen recovered in Tenerife and described by Castillo *et al*.^10^. In fact, Maca-Meyer *et al*.^9^ are of the opinion that there are two species of the “*simonyi* group” in Tenerife: the extinct *G*. *goliath*, and the extant *G*. *intermedia* Hernández *et al*.^11^. The form from La Palma and La Gomera were raised to the rank of species (*G*. *auaritae* and *G*. *bravoana* respectively) but only reasons for the change of the latter were provided by Mateo *et al*.^8^. Subsequently, Martín and Rando^12^ recognised two species in La Gomera: the extinct *G*. *bravoana* and the extant *G*. *gomerana*. Mateo *et al*.^13^, however, showed that only a giant species is present in La Gomera, and that the proper name is *G*. *bravoana*.

We concur with Barahona *et al*.^5^ in that morphological characters supporting the differentiation of many of the species and subspecies of the “*simonyi* group” are problematic in falling within the intraspecific variation of the extant species or being related to size. Moreover, the recognition of new extant species (*G*. *intermedia*) on the basis of soft-tissue characters, or of the validity of *G*. *goliath* on the basis of molecular analyses, complicates the recognition of these taxa in the fossil record, as no clear osteological characters are available for comparison. Finally, the species rank status for *G*. *bravoana* and *G*. *auaritae* as full species have been widely accepted, although reasons for only the former change were properly and formally presented.

We acknowledge that the limit between subspecies and species is on occasions quite subjective. Moreover, once the decision has been taken and widely followed, reversing it may result in greater confusion. The case of giant members of the genus *Gallotia* is especially delicate because the only giant form that is not endangered is *G*. *stehlini*. We have decided to keep the current status of giant taxa as follows, acknowledging that morphological basis for the differentiation of some of these taxa are lacking or are at least problematic: *G*. *stehlini* in Gran Canaria (and introduced in Fuerteventura); *G*. *goliath* (extinct) and *G*. *intermedia* in Tenerife; *G*. *bravoana* in La Gomera; *G*. *auaritae* (extinct, with an unconfirmed sighting, Mínguez *et al*.^14^) in La Palma; and *G*. *simonyi* in El Hierro. For the reasons above, however, we are open to the possibility that all the species of the “*simonyi* group” actually correspond to subspecies of *G*. *simonyi*.

**Figure 1:**
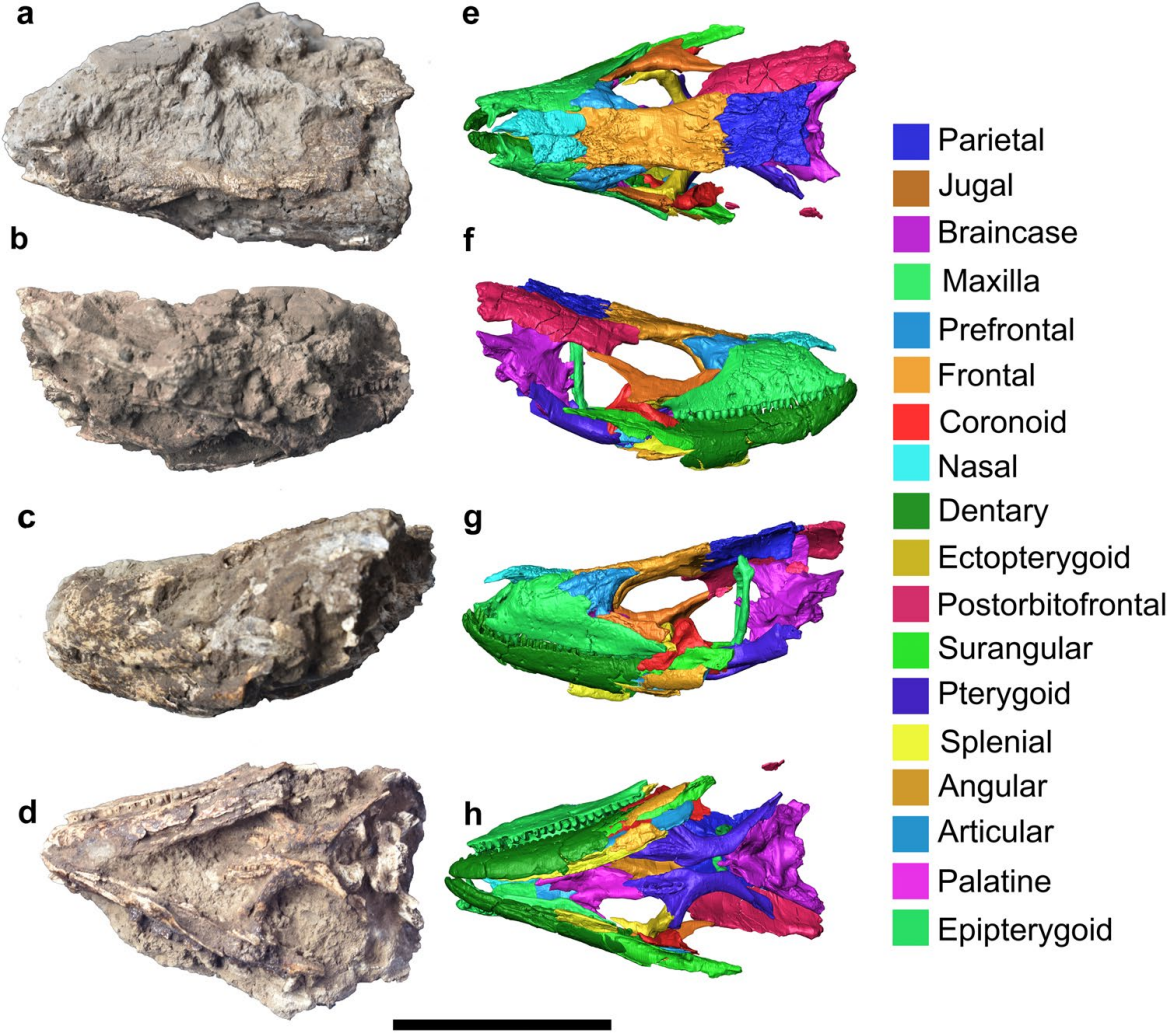
Skull in the matrix (**a**–**d**) and 3D reconstruction (**e**–**h**) of the skull of *Gallotia auaritae* (PCCRULL1169) in left (**a**,**e**), ventral (**b**,**f**), right (**c**,**g**) and dorsal (**d**,**h**) views. Images of the 3D surface in e–h acquired via the screenshot option in Avizo 7.0 (www.avizo3D.com). Scale bar: 5 cm.

**Figure 2:**
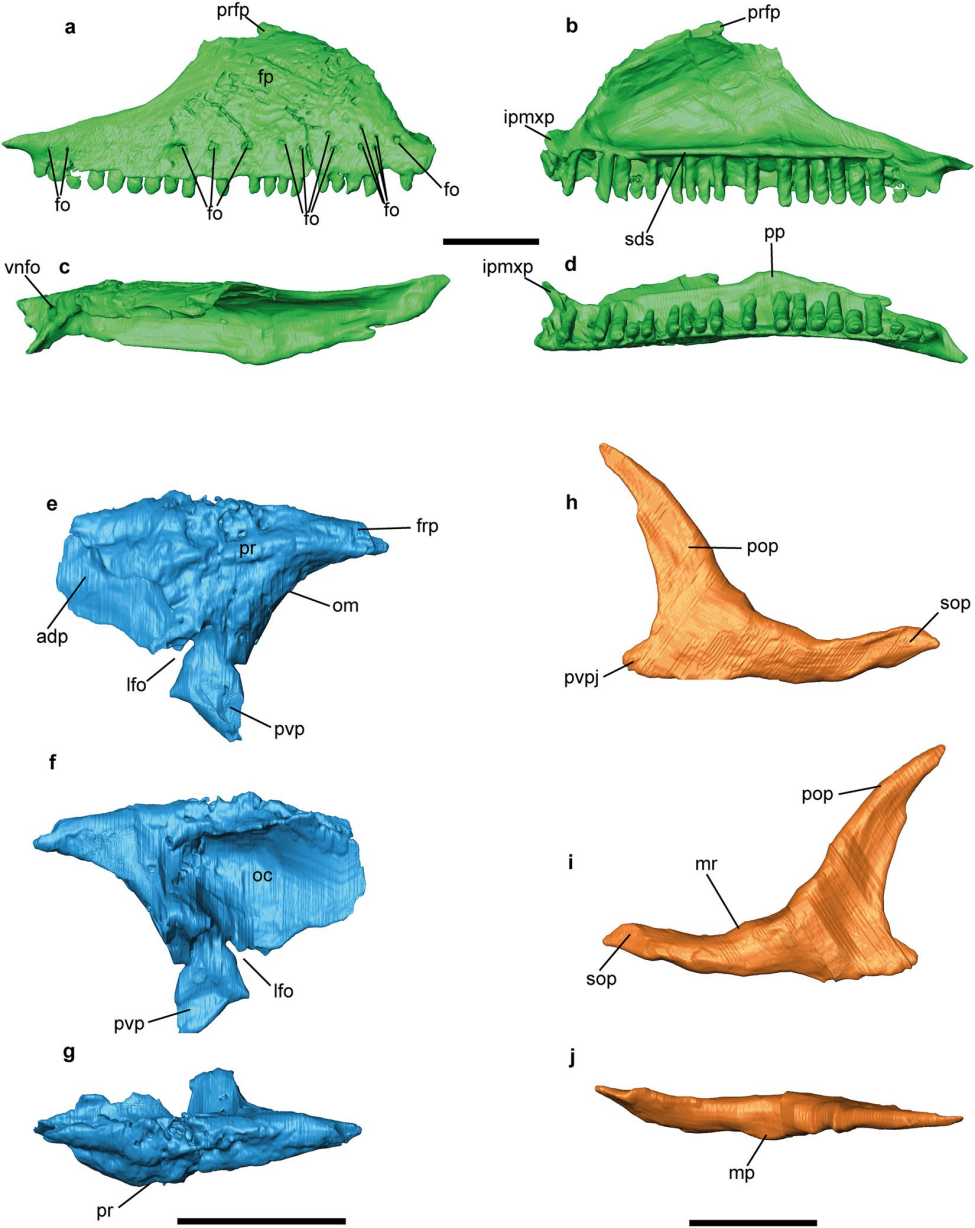
*Gallotia auaritae* (PCCRULL1169) (**a**–**d**) right maxilla in lateral (**a**), medial (**b**), dorsal (**c)** and ventral (**d**) views; (**e**–**g**) left prefrontal in lateral (**e**), medial (**f**) and dorsal (**g**) views; (**h**–**i**) right jugal in lateral (**h**), medial (**i**) and dorsal **(j**) views. Abbreviations: adp, anterodorsal process; fo, foramina; fp, facial process; frp, frontal process; lfo, lacrimal foramen; mr, medial ridge; irpmxp, internal ramus of premaxillary process; mp, medial process; oc, olfactory chamber; om, orbital margin; pop, postorbital process; pp, palatal process; pr, palpebral rim; prfp, prefrontal process; pvp, posteroventral process of the prefrontal; pvpj, posteroventral process of jugal; sds, supradental shelf; sop, suborbital process; vnfo, vomeronasal foramen. Images of the 3D surfaces acquired via the screenshot option in Avizo 7.0 (www.avizo3D.com). Scale bar: 1 cm.

Previously reported fossil specimens assigned to the giant species *G. auaritae* were based on a limited number of elements, but herein we report for the first time an articulated skull (PCCRULL1169; Figs 1 and 2) in an exceptional state of three-dimensional preservation from La Palma island. *Gallotia auaritae* was originally described from cranial and postcranial articulated and disarticulated remains: the holotype corresponds to a dentary and the paratype is composed of a frontal, a parietal and postorbitofrontals, all of them recovered on the Roque de Mazo site (La Palma island; Holocene; Mateo *et al*.^8^). Referred materials correspond to cranial and postcranial bones from La Cueva de los Murciélagos site (Holocene, Los Tilos, La Palma island) and Roque de Mazo site (Holocene; see Mateo *et al*.^8^). The herein reported specimen PCCRULL1169 enables the description of several cranial bones previously unknown for *G. auaritae* (nasal, ectopterygoid, epipterygoid, palatine, angular, splenial; Figs 1 and 2) and allows the observation of the three-dimensional shape of the skull. This skull was located in the sedimentary formation in the Barranco de Las Angustias within of the Caldera de Taburiente (for further details see Suplementary Data 1) from lower-middle Pleistocene. We describe the osteology and the corresponding partial endocast of the fossil specimen thanks to the use of X-ray computed microtomography (Video S1) and provide the first exhaustive phylogenetic morphological analysis including most extant and fossil species of the genus *Gallotia*.

The anatomical nomenclature mainly follows Evans^15^, Rage and Augé^16^ and Klembara *et al*.^17,18^.

## Systematic Palaeontology

SQUAMATA Oppel, 1811

LACERTIFORMES Estes, de Queiroz, and Gauthier, 1988

LACERTIDAE Oppel, 1811

GALLOTIINAE Cano, Báez, López-Jurado, and Ortega, 1984

*Gallotia* Boulenger, 1916

*Gallotia auaritae* Mateo, García Márquez, López Jurado and Barahona, 2001

Figures 1–6

**Figure 3:**
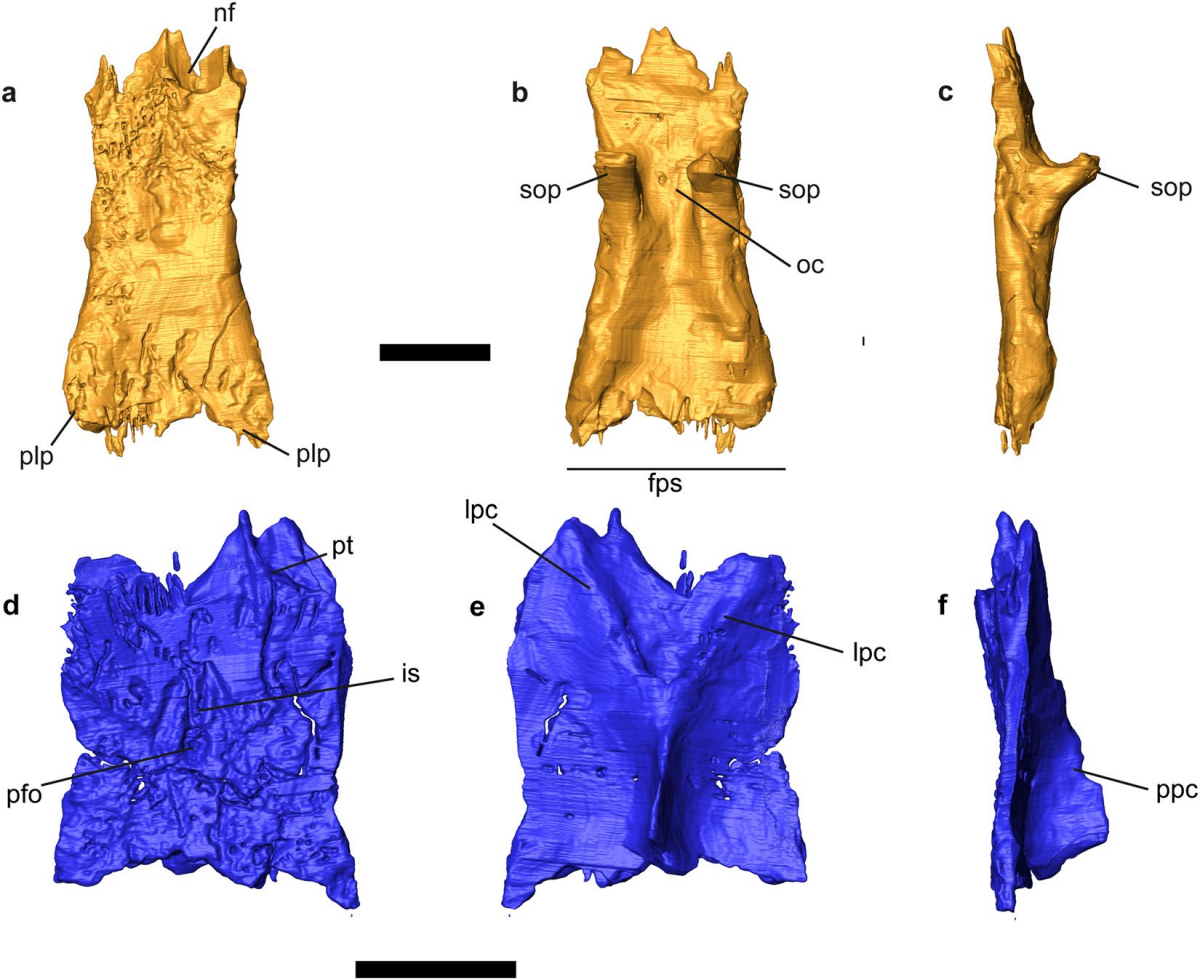
*Gallotia auaritae* (PCCRULL1169) frontal and parietal in dorsal (**a**,**d**), ventral (**b**,**e**) and right lateral (**c**,**f**) views. abbreviations: alp, anterolateral process; amp, anteromedial process; lpc, lateral parietal crest; ppc, posterior parietal crest; fps, frontoparietal suture; is, interparietal shield; nf, nasal facet; oc, olfactory conduct; pfo, pineal foramen; plp, posterolateral process; pt, parietal tab; sop, subolfatory process. Images of the 3D surfaces acquired via the screenshot option in Avizo 7.0 (www.avizo3D.com). Scale bar: 1 cm.

**Figure 4:**
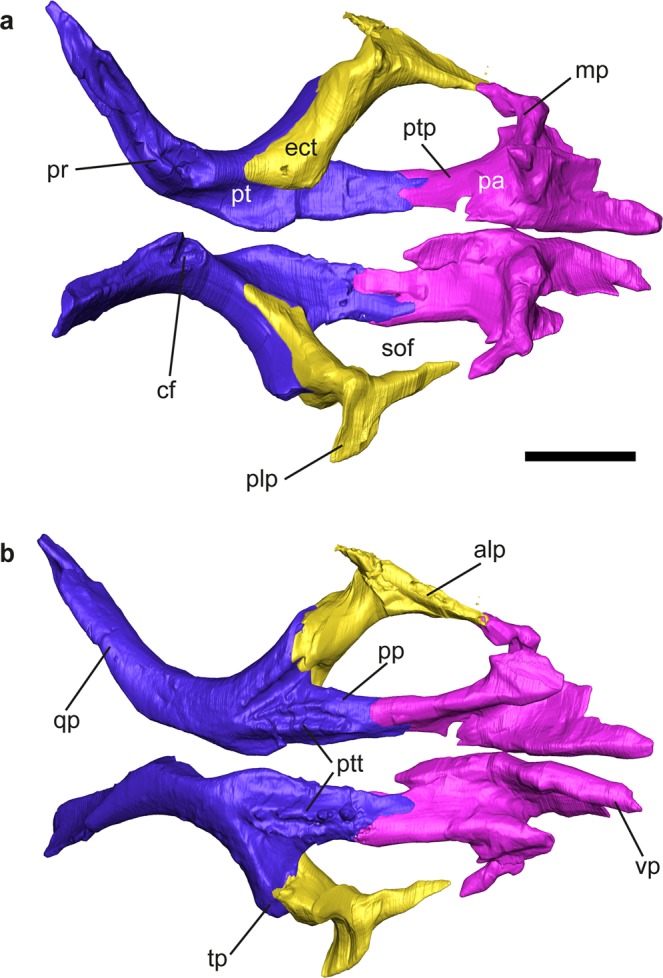
*Gallotia auaritae* (PCCRULL1169) palatine, ectopterygoid and pterygoid in dorsal (**a**) and ventral (**b**) views. Abbreviations: alp, anterolateral process; cf, columenar fossa; ect, ectopterygoid; mp, maxillar process; pa, palatine; plp, posterolateral process; pp, palatine process; pr, pterygoid ridge; pt, pterygoid; ptp, pterygoid process; ptt, pterygoid teeth; qp, quadrate process; sof, suborbital fenestra; tp, transverse process; vp, vomerine process. Images of the 3D surfaces acquired via the screenshot option in Avizo 7.0 (www.avizo3D.com). Scale bar: 1 cm.

**Figure 5:**
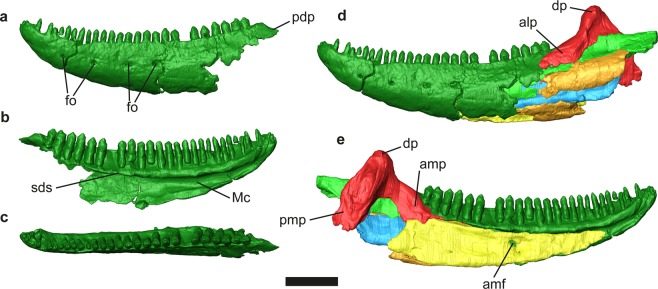
*Gallotia auaritae* (PCCRULL1169) mandible in lateral (**a**), medial (**b**) and dorsal (**c**) views. abbreviations: alp, anterolateral process; amf, anterior mylohyoid foramen; amp, anteromedial process; dp, dorsal process; Mc, Meckelian canal; pdp, posterodorsal process; pmp, posteromedial process; sds, subdental shelf. Images of the 3D surfaces acquired via the screenshot option in Avizo 7.0 (www.avizo3D.com). Scale bar: 1 cm.

**Figure 6:**
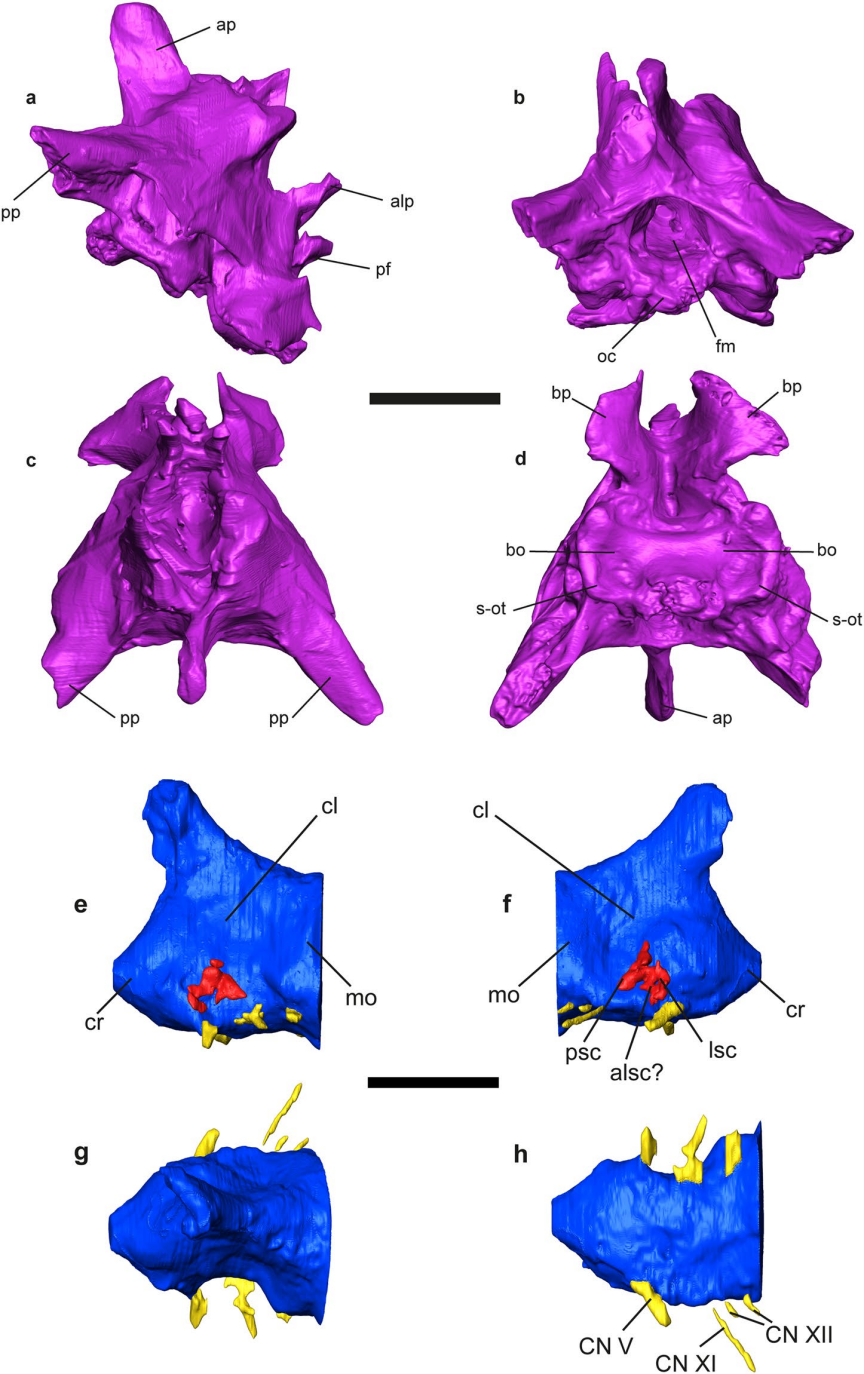
*Gallotia auaritae* (PCCRULL1169) (**a**–**d**) braincase in right lateral (**a**), posterior (**b**), dorsal (**c**) and ventral (**d**) views; (**e**–**h**) cerebrum and inner ear in left lateral (**e**), right lateral (**f**), dorsal (**g**) and ventral (**h**) views Abbreviations: alsc, ampulla lateral semi-circular canal; alp, alar process; ap, ascendant process; bo, basioccipital; bp, basipterigoid process; cl, cerebellum; CN V, nerve V or trigeminal; CN XI, nerve XI or accessory; CN XII, nerve XII or hypoglossal; cr cerebrum; fm, *foramen magnum*; mo, medulla oblongata; lsc, lateral semi-circular canal; oc, occipital condyle; pf, parasphenoid; pp, paraoccipital process; psc, posterior semi-circular canal; s-ot, spheno-occipital tubercula. Images of the 3D surfaces acquired via the screenshot option in Avizo 7.0 (www.avizo3D.com). Scale bar: 1 cm.

**Material:** PCCRULL1169, an articulated skull.

**Locality and Horizon:** Barranco de las Angustias site in La Palma Island (Canary Islands, Spain, Fig. S1); lower-middle Pleistocene, Quaternary.

**Revised Diagnosis:** A giant species of *Gallotia* (with an absolute length of the frontal around or upper to 40 mm, similar to that of *Gallotia goliath*). It differs from all other giant taxa except *G*. *goliath* in the lack of lateral constriction of the frontals (lateral margin of frontals is almost straight). It differs from *G*. *stehlini* in the lack of multicusped teeth (the maximum number of cusps is three, like in the rest of giant forms except *G. goliath* that can reach 4–5 cusps in some teeth. It differs from *G*. *stehlini* (but not from the rest of giant taxa) in presenting two rows of pterygoid teeth forming a V-shape in adults. For a given size, *G*. *auaritae* has more dentary and maxillary teeth than *G*. *simonyi* and *G*. *bravoana*, and less than *G*. *goliath* (see Barahona *et al*.^5^ and Mateo *et al*.^8^).

## Description

### Dermal skull roof

#### Maxilla

Both maxillae are almost complete (Figs 1 and 2a–d). This bone is triangular with an anteroposterior length of 44.3 mm and a dorsoventral maximal height of 14.9 mm. It is in contact with the jugal, the prefrontal and the frontal posterodorsally, and anterodorsally with the nasal and the premaxilla anteriorly. The left maxilla has 23 tooth positions with 19 preserved teeth, whereas the right one has 23 tooth positions with 16 teeth preserved. An ornamented region is preserved in the right labial surface of the facial process of the maxilla and almost 15 labial foramina of different sizes are distributed into one row, except for three of them, that are situated dorsally. A lower number (nine) of labial foramina is present in the left maxilla. The premaxillary process preserves the external and the internal ramus. The external ramus is anteroposteriorly short, robust and its anterior end is upward directed. The internal ramus forms a straight angle with respect the external ramus, is mediolaterally short, robust and its anterior end is forward directed. The maxilla-premaxillary aperture is present. In medial view, the ventral border is straight, form an almost right angle with the dorsal border. In the dorsal surface there is a big vomeronasal foramen. The facial process has a nearly isosceles triangular shape with a convex anterior border and a concave posterior border. Its dorsal portion contains a small hook-shaped process (prefrontal process) dorsoposteriorly directed which contacts with the frontal, as seen in Lacertidae^19^. In medial view, there is a supradental shelf running over almost the entire length of the maxilla (38 mm). The infraorbital foramen (or superior alveolar foramen) is posterior to half of the supradental shelf, it is located at the level of 8th tooth position (counted from posterior). In medial view, the palatal process is located in the posterior middle section of the supradental shelf. It is well developed, and in dorsal view, has an isosceles triangle-shape. The posterior process (or zygomatic process) is long, dorsoventrally short, pointed and does not stepped.

#### Nasal

The nasals are shorter than the frontals (Fig. 1e). The right nasal is the most complete: it is longer (21.8 mm) than wide (7.7 mm). The mediolateral width is constant along the entire nasal length including the posterior process, except in the anteromedial process which is pointed. The medial border of this process is slightly directed to allow space for the nasal process of the premaxilla. The nasal forms the posteromedial border of the exonarina fenestra and presents four foramina forming an anterior convex line. The posterior margin of the two nasals form a W-shape with a notch for the anteromedial process of the frontal in dorsal view.

#### Prefrontal

The prefrontal is triangular in shape and contributes to the anterodorsal border of the orbit and the dorsolateral margin of the lacrimal foramen (anteroposterior length: 18.4 mm; dorsoventral height: 16.1 mm; Figs 1 and 2e–g). It is mediolaterally robust in dorsal aspect. The anterodorsal process is pointed. This process has a smooth anterior zone that articulates with the maxilla. The lacrimal foramen is located between the anterior and the posteroventral processes. The frontal process is short and robust. The orbital margin is widely concave and mediolaterally wide. The palpebral rim is robust and only preserves its dorsal portion, but this character has ontogenetic variation in other species^20^. The posteroventral process is straight and is ventrally directed. It has a tiny process in the posterior margin that is probably broken. The dorsal surface bears an ornamentation (distinct grooves and ridges), whereas the internal surface is smooth and excavated for a wide olfactory chamber.

#### Jugal

This bone is L-shaped (anteroposterior length 24.6 mm; dorsoventral height 19.3 mm; Figs 1e–g and 2h–i). The right jugal is the most complete one. Its external surface is smoothed. The jugal consists of a postorbital and suborbital processes which form the posterior and ventral borders of the orbit, respectively. Both processes are long, narrow and sharp in lateral view. A short and wide posteroventral process is also present. This process is triangular and strongly posteriorly directed. The suborbital process is long and narrow. It broadly overlaps it at the level of the posterior end of the maxillary supradental shelf. The ventral border of the suborbital process is sinuous. This zone contacts with the maxilla in lateral view. On the postorbital process in medial view, there is a medial ridge which is apparently located close to the anterior margin of the bone, creating a broad posterior region behind the ridge. On the suborbital process, the medial ridge apparently runs ventral to mid-height, forming a broad orbital surface. This distribution of the medial ridge corresponds to the jugal type 1 of Čerňanský *et al*.^21^. In dorsal view, the medial process is poorly developed.

#### Frontal

The frontals (Figs 1e and 3a–c) are partially fused and are longer (37.7 mm) than wide (A1, anterior 14.3 mm; A2, half 13.2 mm; A3, posterior 18.9 mm; Fig. S4; Table S2 in Supplementary Data 1) with a trapezium-shape. The anterior margin is transversely broad. The lateral margins of the frontals are slightly concave, and the bone is only slightly narrower anterior to mid-orbit. The end of the prefrontal articulation forms a small step on the lateral margin of the frontal. The postorbitofrontal articulations are located posteriorly. The prefrontals and postorbitofrontals are not in contact, so the frontal is exposed in the orbital margin. The facies nasalis are wide and concave, being the right one the most complete. The anteromedial process is pointed with a triangular shape and extends anteriorly. The posterior tips of the posterolateral processes are broken. The left posterolateral process is the most complete one and, apparently, it slightly projected laterally. The frontoparietal suture is perpendicular to the shaft of the skull. It is slightly concave anteriorly. The dorsal surface presents in its posterior half a depression and a breakage zone related to taphonomic deformation. It is possible to observe the ornamentation in dorsal view. The ornamentation is formed by numerous fine small foramina and grooves. The subolfactory processes are preserved. They are robust, anteroventrally inclined and finger-shaped.

#### Parietal

The parietal table is almost complete (Figs 1e and 3d–f). Only the occipital shield and the supratemporal processes are broken. The parietal is completely covered by ornamented osteodermal shields fused to its dorsal surface. The ornamentation of the parietal table is identical to that of the frontals described above. The parietal table is broken but the preserved fragment is longer (L2: 26 mm) than wide (B1: anterior 16.7 mm; B2: half 19 mm; B3 approximately 16.5 mm; see Fig. S4 and Table S2 in Supplementary Data 1). The mediolateral width B4 cannot be measured because the bone is broken. The lateral sides are slightly concave. The anterior margin possesses two lateral small parietal tabs (only the right one is almost complete), which, as in other lacertoids, underlapped the frontal. The centrally located interparietal shield, bearing the parietal foramen, is small and rhomboidal in shape. The parietal foramen is anteriorly oblique. The interparietal shelf has a rhomboid shape with the parietal foramen in the middle, the lateral shelfs are rectangular and the occipital shelf is not preserved.

In the ventral side the parietal crest forms a Y-shaped crest originating from the anterolateral corners (lateral parietal crests) of the parietal that converge posteromedially (posterior parietal crest). The medial section of the parietal crest is broken before the parietal fossa, but it is deep in the posterior preserved section. This state is present in most modern members of Lacertidae^22^. The parietal foramen penetrates in a cavity formed by the intersection of the crests.

#### Postorbitofrontal

This is a paired bone with a rectangular shape (anteroposterior length 34.1 mm; dorsoventral width 13.3 mm; Fig. 1e,f). An almost complete right postorbitofrontal is preserved, while only the cast and small fragments of bones are preserved of the left postorbitofrontal. The lateral and posterior margins of the right postorbitofrontal are broken. The dorsomedial border is slightly sinuous. The anteromedial and anterolateral processes are partially preserved. These processes form the posterior border of the orbit. The anteromedial process contacts with the posterolateral process of the frontal and the mediolateral process contacts with the dorsal border of the postorbital process of the jugal. In this zone, the postorbitofrontal is smooth and made up by two concave areas, being the dorsal one wider than the ventral one.

### Palatal bones

#### Ectopterygoid

This is a long, slender and triradiated paired bone (Fig. 4). It joins laterally the maxilla and, posteriorly, the pterygoid. In its dorsal surface it has a smoothed area where it articulates with the palatal platform of the maxilla (length 5.5 mm). The posterolateral process is broken. The anterolateral process is anteroposteriorly long and forms the posterolateral margin of the suborbital fenestra.

#### Pterygoid

The pterygoid contributes to create the middle and posterior zone of the palate (Fig. 4). Each pterygoid is triradiate, with palatine and transverse processes anteriorly and a quadrate process posteriorly. The palatine process is long, slender and its lateral and medial borders are parallel. The lateral side forms the medial limit of the suborbital fenestra. Anteriorly, the palatine articulation is forked into two triangular articular surfaces. In both pterygoids there are teeth situated on the ventral surface of the palatine process. These teeth are distributed in two branches forming a V-shape, being the medial branch (eight teeth in the right pterygoid and seven teeth in the left pterygoid) slightly longer than the lateral branch (five teeth in the right pterygoid and four teeth in the left pterygoid); Fig. 4b; see Supplementary Data 1, Table S3). The right pterygoid is more complete. It bears almost seven teeth in the medial branch (the axial end is broken) and five teeth in the lateral branch. The transverse process is robust and extends about two thirds the length of the palatine process. Its anterior end connects with the ectopterygoid. The anteromedial side forms the posterior limit of the suborbital fenestra. The pterygoid notch resembles a V. The quadrate process is anteroposteriorly longer (almost twice) than the palatine process. The palatine process is forwards directed. In the anterior part, the transversal section is quadrate, and the posterior section is mediolaterally narrow. Posterior to the point where the palatine, transversal and quadrate processes unite there is a fossa; the columenar fossa (or epipterygoid fossa), for reception of the epipterygoid (Fig. 4a). Posteriorly to the fossa there is a ridge, the pterigoid ridge (Fig. 4a), where the *M. elevator pterygoide* inserts.

#### Epipterygoid

Both epipterygoids are preserved (Fig. 1g). They are two slender columnar bones that link the parietal with the pterygoid. The proximal end contacts with the alar process of the prootic preserved. The distal ends contact with the pterygoid in the columenar fossa (or epipterygoid fossa) through the pterygoid condyle.

#### Palatine

The palatine is a paired bone (Fig. 4). Both palatines are almost completely preserved. They do not contact at midline. In ventral surface there is a narrow concave area which extends anteroposteriorly conforming the choanal groove (or palatine groove or sulcus palatinus). The palatine has three processes: the maxillary process (lateral), the vomerine process (medial), and the pterygoid process (posterior). The vomerine process is long with a triangular shape, the dorsal surface is concave and in the ventral surface there is a robust medial ridge. The maxillary process is digitiform and lateroposteriorly directed. In the ventral surface there is a robust and mediolaterally wide tap. This ridge is extremely developed anteriorly covering the infraorbital foramen which is not complete, lacking the posterior border. The Nerve V (maxillar nerve) runs through the infraorbital foramen pass. In the dorsal surface and perpendicular to the sagittal axis is a strongly developed palatine crest; it is dorsoventrally high and has a globose area in its medial margin. This crest separates the anteromedial and anterolateral processes. The pterygoid process is almost as long as the anteromedial process and is narrow and rectangular in shape.

#### Mandible

None of the mandibles is complete because their most posterior portion is broken in both cases (Figs 1f–h and 5; Supplementary Data 1, Table S3). The preserved portion has an anteroposterior length of 69.1 mm. The dentary, the splenial, the coronoid, the angular, the surangular and the articular bones are preserved.

#### Dentary

Both dentaries are almost completely preserved, except for their most posteroventral portions (Figs 1f–h and 5). They have an approximately anteroposterior length of 48 mm as preserved. The dorsal border is concave, whereas the ventral border is convex and slightly curved medially at its anterior end. The transversal section is C-shaped. The lateral surface is pierced by a series of at least 5 labial foramina, distributed in straight line along the half line of the dentary (to the alveolar lower nerve of the cutaneous branch of the mandibular nerve III?). All of them have an oval shape and a similar size. In both dentaries the posterodorsal process is almost completely preserved, whereas the posteroventral process is broken (Fig. 5a,b,d). The posterodorsal process apparently finishes at the level of the anterior half of the dorsal process of the coronoid. The posterodorsal and posteroventral processes are separated by an anterior wedge-shaped extension of the surangular bone in lateral view. The medial side of the dentary is concave and presents the subdental shelf with a length 44.4 mm measured between the most proximal end of the dentary and the last tooth of the dentary (Figs S4c and 5b). The subdental shelf sharpens posteriorly. The subdental shelf stands between the alveolar platform (situated dorsally) and the Meckelian canal (situated ventrally). Both dentaries bear 27 teeth positions with 24 and 21 preserved teeth in the left and right dentary, respectively. The Meckelian canal is deeply excavated, open in its entire length, anteriorly narrow and posteriorly wide (Fig. 5b).

#### Coronoid

 Both coronoids (Fig. 5d,e) are almost completely preserved, being the left one the most complete. In medial view, the coronoid has the shape of an inverted chevron. The coronoid presents four complete processes: anterolateral, anteromedial, posteromedial and dorsal. The dorsal process is robust and vertical. In lateral view, it presents a slightly convex ridge in the middle while posteriorly there is a strongly concave area. The anterolateral and the fragment of anteromedial processes overlap laterally and medially the posterior end of the dentary, respectively. In lateral view, the anterolateral process is long and has a rectangular shape. In medial view the anteromedial process in articulation with the splenial is subtriangular. The anteromedial process surpasses the last tooth position anteriorly. In medial view the posteromedial process is rectangular with a slightly concave medial surface and a marked ridge in its anterior border.

#### Angular

Only two fragments of the left angular are preserved (Fig. 5d). These fragments are anteroposteriorly elongate and laterally convex.

#### Surangular

The surangular is anteroposteriorly long (41.7 mm) with a rectangular shape in lateral view (Fig. 5d). It is located posterior to the dentary and forms the dorsal border of the posterior part of the mandible. The anterior end is broken, and the dorsal margin is straight in lateral view. The upper half of the lateral side is slightly concave whereas the lower half is convex. _In medial view, posteriorly, in the upper border to half of its length, the bone has a dilated zone (dorsoventral height 5.4 mm). The posteromedial process of the coronoid is located above the posterior part of the dilated zone. The dorsal border presents a shelf which is posteriorly wider than anteriorly, in medial view.

#### Articular

 An almost complete left articular and a fragment of the right articular are preserved. The anterior process, with a tongue-like morphology, and the anterior margin of the dorsal process are preserved in the left articular (Fig. 5c,d). The medial side is straight and in the lateral side there is a ridge that projects laterally. The preserved fragment of the ridge is mediolaterally narrow.

#### Splenial

It is a long, flat and triangular bone that covers the Meckelian canal (Fig. 5e). In the medial side is the anterior mylohyoid foramen. In lateral view is the prearticular crest of the splenial separates two areas, the dorsal area being bigger than the ventral one.

#### Dentition

The dentition is pleurodont and heterodont (Fig. 5). Teeth are conical. It is not possible to distinguish the exact number of cusps in the 3D model. However, direct observations in the left dentary of the specimen, which is not so extensively covered by the matrix, allow an assessment of the morphology of some teeth. The most anterior teeth are monocuspid or bicuspid, and from the thirteenth position sometimes have an incipient third cusp.

### Braincase

#### Supraoccipital

This bone delimits the dorsal margin of the *foramen magnun* (Fig. 6b,c). In dorsal view, it has apparently a hexagonal shape. The ascendent process is large and located in the middle of the supraoccipital. In lateral view, this process is slightly directed backwards. It has a rectangular shape and its anterior and posterior margins are straight and parallel.

#### Prootic

This bone forms the lateral side of the neurocranium and the posterolateral side of the cranium (Fig. 6a,c). It covers part of the occipital fossa and the interfenestra and tubular ridges. Part of the prootic crest in lateral view is possibly preserved. This ridge is robust and anteroposteriorly directed.

The prootic bears three processes: the anterodorsal or alar, the anteroventral and the posterior processes. The alar and anteroventral processes are not preserved. The posterior process is strongly fused with the paraoccipital process so that it is not possible to differentiate both bones.

#### Otooccipital

This bone is the result of the fusion of the exoccipital and the opisthotic (Fig. 6a). This bone forms the lateral margin of the *foramen magnum* and it is concave in posterior view. It is fused with the supraoccipital and it is not possible to observe the suture between them. The paraoccipital processes are not complete; their posterior ends are broken. These processes are robust and with a triangular shape transversal section. In the base of the processes there are foramina for the nerves IX and X in lateral side.

#### Sphenoid

This bone, together with the basioccipital, form the floor of the neurocranium (Fig. 6d). It is not possible to observe the suture between them. The basal tubercles are well developed, thick and rounded. In ventral view, it is quadrangular and bears two extremely developed basipterigoid processes. These processes are wing-shaped with the lateral margin convex and they are ventrally in ventral view. In lateral view, the basipterigoid processes are hook-shaped with the anterior margin straight and the posterior border sharp. In anterior view, the parasphenoid is located between the basipterigoid processes and the alar processes. It is short, robust, finger-shaped and anteriorly directed, but its anterior end is broken. Above this process are the alar processes of sphenoid. These are slender and resemble a finger shape. The parasphenoid does not exceed anteriorly the length of these processes. A foramen for the nerve VI is located at the base of each alar processes, and under them is a foramen for nerve VII or the carotid artery. The ventrolateral ridges join thoroughly with the spheno-occipital tubercula.

#### Basioccipital

The basioccipital is incomplete, the posterior portion which contributed to the occipital condyle is broken, so that it is not possible to observe its shape. The fragment preserved is rectangular, being mediolaterally wider than anteroposteriorly long and concave in ventral view (Fig. 6a,d). The spheno-occipitals tuberculae are well developed and posteromedially directed.

### Soft tissue

#### Cerebrum

The most posterior part of the endocast has been reconstructed (Fig. 6e–h). This corresponds to the otic-occipital region. A small part of the cerebrum, the cerebellum and the beginning of the medulla oblongata, is located in this region, the rest of it being in the cartilaginous region of the orbitotemporal and not preserved. The preserved portion is anteroposteriorly short (8.3 mm) and has a subrectangular shape in dorsal view. The zone of the medulla oblongata has a circular cross section, while the cerebellum zone is anteriorly sharpened and considerably mediolaterally constricted with a triangular cross section. The cerebrum zone is slightly mediolaterally wider than the cerebellum, but less than the medulla oblongata. In both laterals of the cerebellum there is a strong concave area situated in the upper portion. In the dorsal side there is a slightly anteriorly direct channel (angle 46.3° with respect anteroposterior axis of the cerebrum). It is anteroposteriorly long and mediolaterally narrow. It has been possible to reconstruct the nerves V (trigeminal), XI (accessory) and XII (hypoglossal) in the right side.

#### Inner ear

The right inner ear is the most complete one (Fig. 6e,f). It preserves the posterior and the lateral semi-circular canal and possibly the ampulla of the lateral semi-circular canal. They are wide and short.

### Phylogenetic analysis

**Figure 7:**
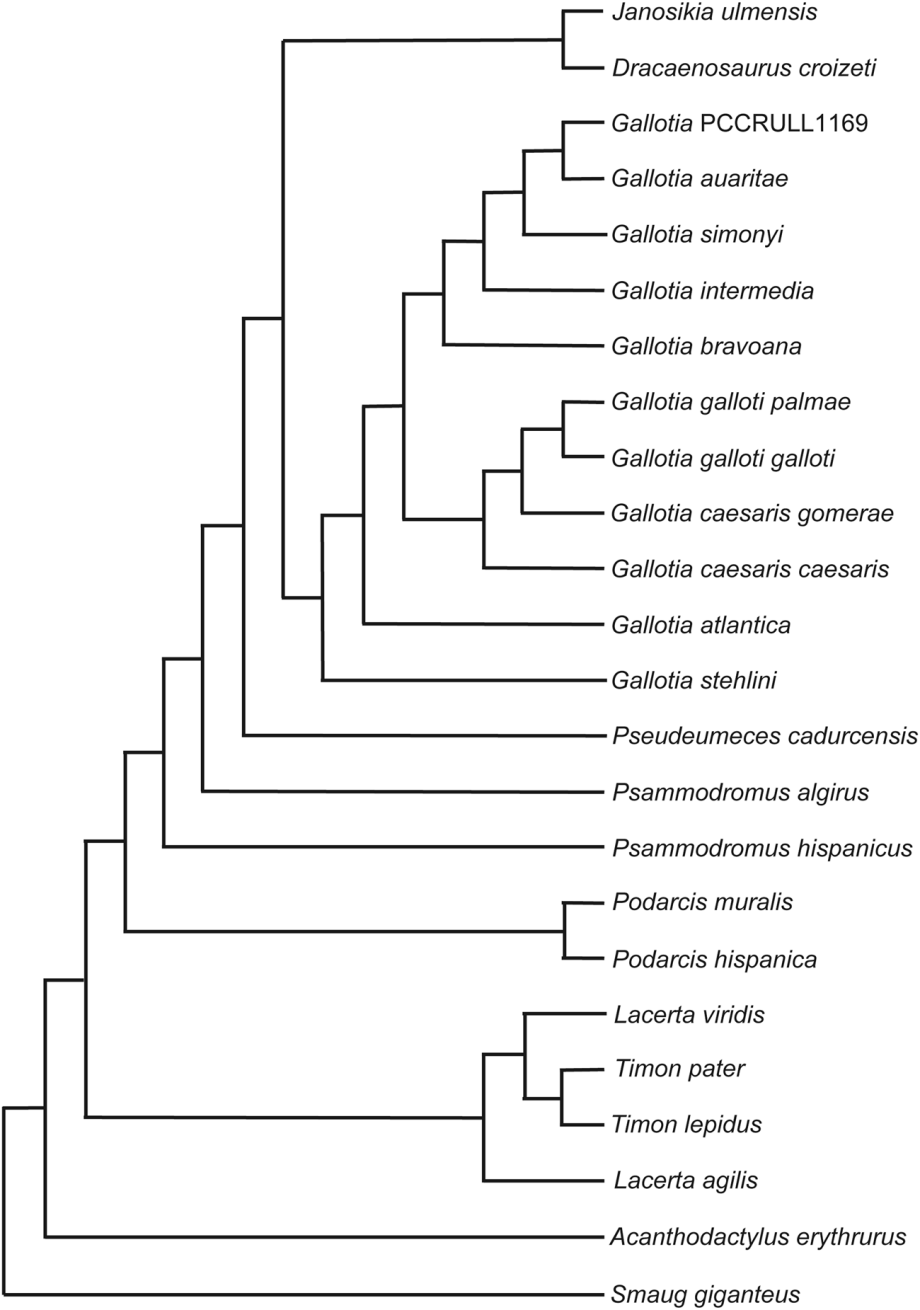
One of the three most parsimonious trees recovered in the ordered and weighted analysis. Note that the sister group relationship between the coding of PCCRULL1169 and *G. auaritae* is congruent with a referral of the former to the latter.

Our analysis with a selection of characters ordered (see Methods) resulted in three most parsimonious trees (MPT’s) with a length of 140.77 steps (CI = 0.450, RI = 0.68, RC = 0.31; Fig. 7; Supplementary Data S5). The resulting overall topology for the main analysis is very similar to the one recovered by Čerňanský *et al*.^23^: Lacertinae is paraphyletic, Gallotiinae is monophyletic and includes *Psammodromus* and three fossil genera (*Janosikia, Dracaenosaurus* and *Pseudeumeces*) in an intermediate position between it and *Gallotia*, which is monophyletic. The recovered topology for the genus *Gallotia* approaches the one recovered by molecular studies (e.g. Cox *et al*.^24^): 1) *G. stehlini* is the most basal species, followed by *G. atlantica*. Sister to *G. atlantica* there is a large clade formed by *G. galloti* (two subspecies) and *G. caesaris* (two subspecies) on one side, and *G. simonyi* and species closely related (the “*simonyi* group”), containing *G*. *simonyi*, *G*. *intermedia*, *G*. *bravoana* and *G*. *auaritae*. Note that *G*. *goliath* is not included in our main analysis because the few codifiable characters were redundant with the coding of *G*. *auaritae*. The results of an additional analysis including *G*. *goliath* are reported and discussed in the Supplementary Data S3, S6, S7). In our main analysis, the specimen here reported from La Palma nests inside the *G*. “*simonyi* group”, more specifically as sister taxon to *G. auaritae*, suggesting that might be conspecific to it. This interpretation fits well with the fact that *G. auaritae* is the only giant *Gallotia* species from La Palma. The “*simonyi* group” is characterized by the following synapomorphies: character 1, skull size (1– > 2), highlighting an increase of skull size in the “*simonyi* group”; character 5, angle of nasal process of premaxilla (1 ==> 0), this is a change from a more oblique to a more vertical process of this bone; character 7, premaxillary teeth in adults (2– > 3), recording a change from a tooth number between seven and eight to a tooth number of nine in the premaxilla; characters 13 (maxillary tooth size: 1 ==> 0) and 14 (maxillary tooth count: 0 ==> 3) reveal, respectively, a change from a low number of maxillary teeth of increasing size to a much higher tooth number of equal size; character 25, frontals (1 −> 0), corresponding to a change from partially fused to discrete frontals; and finally, character 34, a parietal that extends over braincase in dorsal view (1 ==> 2). The lack of mediolateral constriction (character 26, state 0) is the only unambiguous synapomorphy uniting the fossil from La Palma and *G. auaritae* (Supplementary Data 4). However, the change in character 14 (number of maxillary tooth size), which is coded differently in the fossil specimen herein reported (coded as 2) and *G. auaritae* (and in fact, the rest of species closely related to *G. simonyi*, which are all of them coded as 3), is probably related to the fact that we used the maximum tooth number ever recorded for these species (corresponding to very large specimens). The fossil specimen from La Palma present a minimum number of 23 teeth but we cannot assess with confidence if it was fully grown, so it is possible that the species could have attained a larger size, adding some more teeth to the maxilla and thus, reaching the minimum number (25 maxilla teeth) to be scored 3. Moreover, it is not clear if a few additional teeth might have been present in preserved or missing portions of the maxilla. In any case, total tooth number in the dentary and maxilla in giant members of *Gallotia* depend on the size of the specimen (Barahona *et al*.^5^; Mateo *et al*.^8^), and thus of little help unless specimens of the same size are compared. For a full list of synapomorphies of all nodes see the supplementary information.

## Discussion

According to Oliver *et al*.^25^ the regions with complex geological histories, such as a volcanic archipelago like the Canary Islands, often have diverse and highly endemic biotas^26,27^. The high endemicity of the Canary Archipelago is related to their volcanic oceanic origin, isolation and varied topography, which have influenced the processes of evolution and speciation^28^. These factors and the absence of predators shaped the evolution of the lizards of the genus *Gallotia*.

The pattern of colonization of the genus *Gallotia* shows a distribution from east to west where the giant taxa are found in the western and younger islands^9,29^. According to Čerňanský *et al*.^30^ a European ancestor of *Gallotia*, which had already achieved a large body size, arrived at the archipelago with the first migratory flow. Once the ancestor arrived at the Canary Islands (approximately 17–20 Ma^24,30^) it gradually colonised all islands, although the pattern and timing of dispersion is poorly understood. In any case, *Gallotia* reached La Palma at some point after its formation 1.77 Ma, and there the endemic species *G*. *auaritae* evolved. The discovery of the fossil articulated skull PCCRULL1169 allows for the first time the description of multiple cranial and mandibular bones previously unknown for *G. auaritae*. This specimen allows direct comparisons with the rest of giant fossil and extant taxa of the genus *Gallotia*. All these new data contribute to our knowledge of the range of inter- and intraspecific variation in the osteology of the giant species of the genus *Gallotia*. The newly reported specimen referred to the giant lizard taxon *G. auaritae* has a total skull length of approximately 90 mm (Figs 3 and 4), being slightly smaller than *G. goliath* in size, three times bigger than *G. bravoana*, double than *G. intermedia* and a third bigger than *G. simonyi* and *G. stehlini*^10^. This skull would correspond to an adult with an estimated maximum snout-vent length (SVL) of approximately 380 mm, a similar size to the holotype, according to Mateo *et al*.^8^. For the anatomical comparison of the skull of *G. auaritae* (PCCRULL1169) with extant *Gallotia* taxa see Supplementary Data 1.

The endemic Canarian genus *Gallotia* represents a great opportunity to investigate evolutionary processes and patterns happening in oceanic islands. Specifically, evolutionary paths followed by different taxa in different islands towards distinct degrees of gigantism or adaptations to herbivory are extremely interesting phenomena that deserve attention. Moreover, a simple better understanding of the taxonomy of the genus may lead to a better managing of the species conservation plans. This is especially important regarding the giant taxa because they are, in general (an exception is *G. stehlini*), more endangered than small species. Giant forms of *Gallotia* are distributed along the most occidental islands (Gran Canaria, Tenerife, La Gomera, El Hierro and La Palma) with five species described. Both *G. auaritae* (including the fossil specimen described herein) and *G. goliath* present the biggest skulls, which have frontals anteroposteriorly longer than parietals, unlike in *G. bravoana*, in *G. stehlini* and in *G. simonyi* (see Supplementary Data 1, Table S2). Only a few disarticulated cranial bones of *G. auaritae* were previously known, so the newly reported specimen is the first known almost complete articulated skull for this species. A new phylogenetic analysis including all extant species of the Canary Archipelago and the new specimen has recovered a monophyletic *Gallotia* clade with similar topology to that of molecular analyses. The described specimen shows the almost straight lateral margins of the paired frontals described in the holotype of *G*. *auaritae*, and some characters of the group formed by the species *G. auaritae, G. bravoana, G. intermedia* and *G. simonyi*: 1) big skull (between 20–40 mm), 2) maxillary tooth crown size and height constant throughout tooth row, 3) presumably a maxillary tooth count of more than 25, 4) frontals unfused, and 5) occiput fully covered by the parietal, or nearly so, in dorsal view. Most of these characters would presumably apply to *G*. *goliath* too, although this taxon was not included in the phylogenetic analysis. Our study adds new important data to the poorly known morphology of *G*. *auaritae*, and the performed phylogenetic analysis reveals an unexpected power of resolution to recover a well resolved phylogeny of the genus *Gallotia*, and for inferring the phylogenetic position of complete enough fossils of the genus, helping thus in their identification. This will be especially important in identifying fossils of species currently absent from the island where they are found.

## Methods

### Institutional abbreviations

*DBAEYGULL*, Department of Animal Biology, Edaphology and Geology, Universidad de La Laguna (Tenerife, Canary Islands, Spain); *DBULPGC*, Departament of Biology, Universidad de Las Palmas de Gran Canaria (Gran Canaria, Canary Islands, Spain); *DZULL*, Departament of Zoology, Universidad de La Laguna (Tenerife, Canary Islands, Spain); *MUNA*, Museo de Naturaleza y Arqueología (Tenerife, Canary Islands, Spain); *PCCRULL*, Paleontology Collection- Carolina Castillo Ruiz, Universidad de La Laguna (Tenerife, Canary Islands, Spain); *UMCG*, Unidad de Medioambiente del Cabildo Insular de la Gomera (Canary Islands, Spain); *UMCIH* Unidad de Medioambiente del Cabildo Insular de El Hierro (Canary Islands, Spain).

### Material and X-ray computed microtomography

The studied specimen (PCCRULL1169) is stored at the Paleontological unit (Área de Paleontología) of Universidad de la Laguna from Tenerife (Canary Islands, Spain) and corresponds to an almost complete skull mostly covered and infilled by volcanic matrix. The specimen was recovered from La Palma Island (Canary Islands, Spain, Figs 1 and 2) by C. Castillo Ruiz, J.R. Colmenero, J. De La Nuez and R. Casillas in 2000.

The specimen was scanned at the Multidisciplinary Lab (MLAB) of the “Abdus Salam” International Centre for Theoretical Physics (ICTP, Trieste, Italy), using X-ray computed microtomography (microCT). For further details regarding the equipment see Tuniz *et al*.^31^. The parameters used are presented in Supplementary Data S1. For anatomical comparisons and, particularly, for the phylogenetic analysis, several specimens from extant species (see below) were also scanned at the same institution. See Supplementary Data S2 for the parameters used in each case. Raw data from each scan was imported (as stack of TIFF 8-bit files) to Avizo 7.0 to generate a 3D surface from the microCT images, digitally extracting the specimen from the surrounding matrix (similarly to Bolet *et al*.^32^, Holgado *et al*.^33^) and to deep in the neuroanatomy of the fossil specimen (in similar manner to Cruzado-Caballero *et al*.^34^). Images of the 3D surface in Figs 1–6 were acquired via the screenshot option in Avizo 7.0.

### Comparative specimens used

Specimens of the following extant and fossil species of *Gallotia* were microCT scanned at the International Centre for Theoretical Physics (ICTP, Trieste, Italy): *Gallotia atlantica*, *Gallotia bravoana*, *Gallotia caesaris caesaris*, *Gallotia caesaris gomerae*, *Gallotia galloti galloti*, *Gallotia galloti palmae*, *Gallotia intermedia*, *Gallotia simonyi* and *Gallotia stehlini*. See Supplementary Data S2 for specimen details and microCT scan setups. All these specimens were studied to perform the phylogenetics analysis, but comparison was mainly focused on the giant species (*G. bravoana, G. goliath, G. simonyi* and *G. stehlini*). *Gallotia auaritae* material was not scanned, but original material was accessed and photographed.

### Phylogenetic analysis

Our analysis is based on the matrix of Čerňanský *et al*.^23^ which, in turn, is a modified version of the matrix published by Čerňanský *et al*.^30^. Only two species of *Gallotia* were coded in Čerňanský *et al*.^23^: *Gallotia stehlini* and *Gallotia galloti*. We added nine taxa (*Gallotia atlantica*, *Gallotia bravoana*, *Gallotia caesaris caesaris*, *Gallotia caesaris gomerae, Gallotia galloti galloti, Gallotia galloti palmae, Gallotia intermedia, Gallotia simonyi, Gallotia auaritae*) to the matrix, and we replaced the coding of *Gallotia galloti* by two codings at the subspecies level (*Gallotia galloti galloti, Gallotia galloti palma*e) (Supplementary Data S3a). We also modified the coding of *G. stehlini*, and we added a coding for the fossil specimen from La Palma. The resulting matrix has 25 taxa and 64 characters, being the genus *Gallotia* represented by 12 units at the species or subspecies level (plus the fossil specimen under study). We also run an analysis including *G*. *goliath* (see Supplementary Data S3, S3b). All analysis were run in PAUP 4.0a159^35^, with the same settings as reported in Čerňanský *et al*.^23^: 19 characters were treated as additive, ordered under between character weighting^36^. Multistate taxa were treated as polymorphisms. We ran a heuristic search algorithm with tree bisection and reconnection (TBR) branch swapping, and random-addition sequence set to 10000 replicates, with other parameters set to default values.

## Supplementary information

Supplementary Information

Supplementary Information

Supplementary Information

Supplementary Information

Supplementary Information

Supplementary Information
