# Supplementary material for: First nearly complete skull of Gallotia auaritae (lower-middle Pleistocene, Squamata, Gallotiinae) and a morphological phylogenetic analysis of the genus Gallotia
Source: Sci Rep. 2019 Nov 12;9:16629. doi: 10.1038/s41598-019-52244-z (PMC6851374; doi:10.1038/s41598-019-52244-z)
Supplement: Supplementary file 1 — Supplementary Information [file 41598_2019_52244_MOESM1_ESM.pdf]

First nearly complete skull of *Gallotia auaritae* (lower-middle Pleistocene, Squamata, Gallotiinae) and a morphological phylogenetic analysis of the genus *Gallotia*

Penélope Cruzado-Caballero, Carolina Castillo Ruiz, Arnau Bolet, Juan Ramón Colmenero, Julio De la Nuez, Ramón Casillas, Sergio Llacer, Federico Bernardini, Josep Fortuny

Supplementary Data S1

Characteristics of the fossil record in volcanic active island

The fossil record from the Canarian Archipelago (table S1) is particularly rich mainly on volcanic tubes and, to a lesser extent, on paleodunes and volcanic lapilli deposits. In most cases the remains have been found disarticulated and represent material of several specimens of different ontogenetic stages. Exceptional cases in terms of preservation are the specimen here studied (PCCRULL1169) which is a unique fossil in sandstones/siltstones sediments, the mummy referred to *G. goliath* from Tenerife island [10] or four lapilli blocks recovered from Gran Canaria island [37] with complete and partially complete skeletons of four specimens referred to *Gallotia* aff. *stehlini*. Additional articulated specimens from the latter locality are currently under study [38].

Geological setting

La Palma is the northwestern most island of the Canary Islands archipelago and, according to the age of its subaerial volcanism, its sediments are less than 1.7 My old. Along with the adjacent island of El Hierro, La Palma island is in an early evolutionary stage (shield building phase [39]).

The Caldera de Taburiente is the most striking geomorphological feature, that consists of an almost circular amphitheatre built by volcanic, subvolcanic and erosive processes.

It is situated in the interior of the Northern Shield, with a diameter of ca. 9 km, 950 m-high subvertical walls, and a depth of 1500 m. This is connected with the eastern Atlantic coast of the island through the Barranco de Las Angustias, a narrow and deep canyon that flows into the Atlantic Ocean at Tazacorte (Fig. S1). These are sediments composed of Las Angustias Breccias and Conglomerates (Unit IV of Colmenero et al. [40]), and are forming the margins of the Barranco de las Angustias.

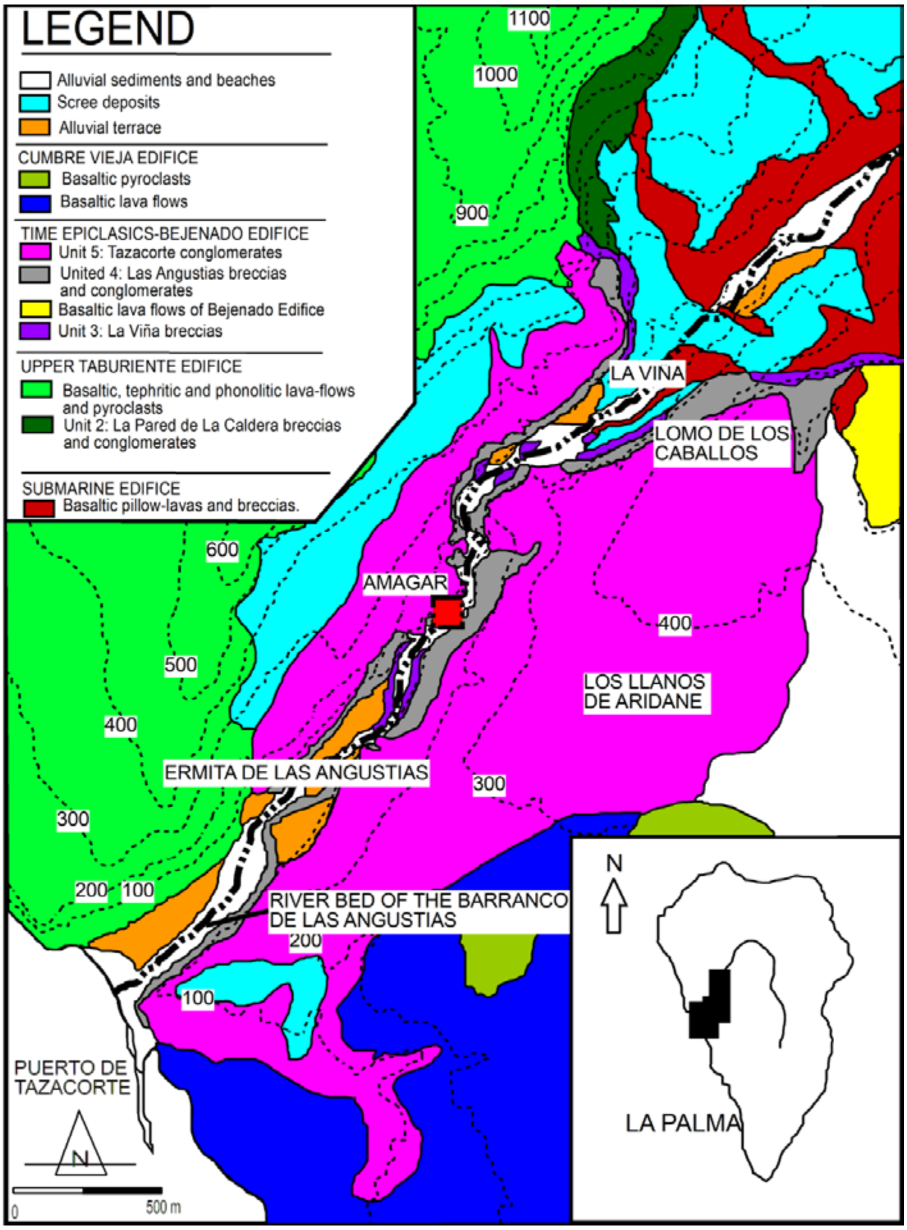

Figure S1. Map showing the location of the Barranco de las Angustias site (La Palma, Canary island, Spain).

Stratigraphy and sedimentology of the succession

As shown in Fig. S2, the succession containing lizard fossil remains corresponds to a 9 m thick outcrop mainly composed by alternations of silty sands, silts and clays forming normally graded stacked sequences, interbedded with several levels up to 1.5 m thick conglomerates beds. The levels of very fine sand with sandy and clayey silts are often brecciated, forming horizons of autoclastic breccias, with a similar appearance to that of the desiccation polygons. Frequent traces of roots are also present. These dispersed within these fine levels pebble to boulder-sized clasts (up to 60 cm in diameter) appear, arranged in discontinuous levels or isolated in the mudstones.

The conglomerate beds are mainly poorly-to-very poorly sorted, clast-supported, made of rounded to subangular pebbles to cobbles, and set in a fine-gravel-to-coarse-sand grade matrix. The beds are mostly lenticular and show overall erosive bases and a crudely normally graded trend. Laterally, they wedge out into the fine-grained intervals.

Very similar deposits to the previous ones have been described upstream of Barranco de Las Angustias (275 meters high) by Vegas [41] and García-Romero et al. [42], as the Caldera de Taburiente lacustrine unit (CTL Unit). They are mainly composed of mudstones with a minor proportion of litharenites (volcarenites) and conglomerates, and have been interpreted as turbidites and clays deposited in a lacustrine environment. In some layers there are fluid escape structures and plant fossils (leaves, stems and seeds). The macroflora fossil record suggests an age of lower-middle Pleistocene [43]. However, taking into account that the sedimentary sequence of the conglomerates and breccias of El Time is located over the basaltic flows of the Bejenado Massif, dated between 0.49 and 0.56 myrs, this sedimentary sequence must be less than 0.49 myrs old, or even less than 0.4 myrs old [40]. Both successions probably represent parts of a relatively ephemeral

shallow lake developed in the Barranco de las Angustias behind a natural dam formed as a consequence of landslides that would sporadically close the natural flow of water in the ravine, during the lower-middle Pleistocene. The presence of soft sedimentary structures in the successions reveals that very frequent earthquakes accompanied the sedimentation.

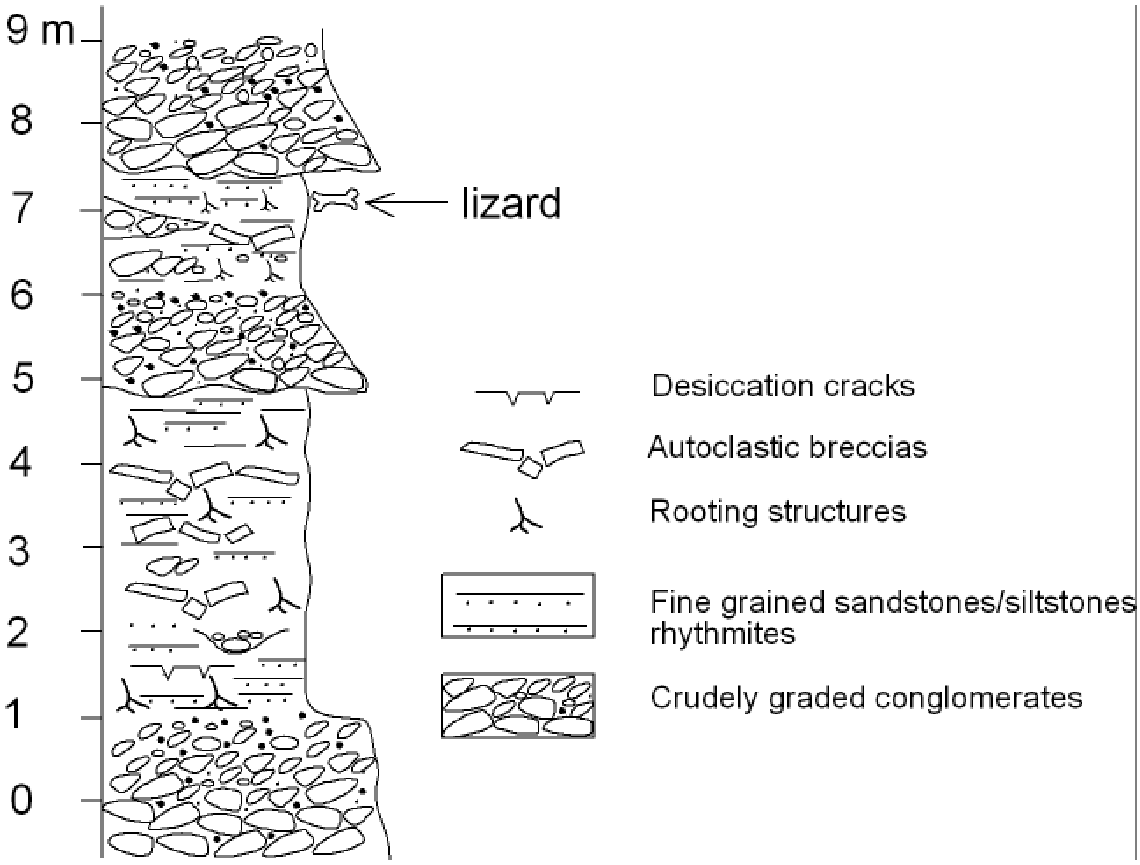

Figure S2. Barranco de las Angustias stratigraphic sections (La Palma, Canary island, Spain), with the location of the skull of *Gallotia auaritae*.

Comparison of cranial elements

When comparing the skulls of the different giant species of *Gallotia* several differences in the development and shape of some bones and processes are evident. These include the anterodorsal process of the prefrontal or the posteroventral and medial processes of

the jugal. The comparisons of the herein reported specimen were made fundamentally with a jaw (type) and cranial roof (paratype) of *G. auaritae*, but also elements the fossil giant species *G. goliath* and extant giant species: *G. stehlini, G. simonyi*, *G. bravoana* and *G. intermedia*.

- Maxillae

The anteroposterior length of this bone in the fossil is bigger than that of the extant giant specimens: it is twice as long as in *G. bravoana* and *G. intermedia* and one third longer than in *G. simonyi* and *G. stehlini* (see supplementary table S3).

- Jugal

The posteroventral process of the jugal shows different shapes among giant species: in *G. bravoana* it is triangular and is poorly developed. A similar morphology is also present in *G. stehlini, G. simonyi* and the herein reported specimen. However, in *G. stehlini* this process shows a greater posterior projection and a slenderer shape. In contrast, in *G. intermedia* the posteroventral process is quadrangular, robust and slightly posteriorly projected. In dorsal view, according with the specimens scanned the medial process is poorly developed in *G. intermedia, G. bravoana* and in the reported fossil specimen and it is small but well defined in *G. simonyi*, whereas in *G. stehlini* this process is absent.

- Frontal

In adults of small and giant species (*G. galloti, G. caesaris, G. atlantica, G. intermedia, G. stehlini* [30]) and possibly in *G. simonyi* and *G. bravoana* (pers. obs. P.C-C), there is a tendency towards frontals fusion. The length of the frontal in the reported specimen is similar in size to other fossils of giant species such as *G. goliath* and slightly smaller

than to the holotype of *G. auaritae* (see table S2). This length is approximately between two and three times bigger than in *G. bravoana, G. stehlini, G. simonyi* and *G. intermedia* (see table S2). The lateral margins of the frontal show two concave areas in *G. intermedia, G. simonyi* and *G. stehlini,* unlike in the fossil and *G. auaritae* and *G. goliath*.

- Parietal

The anteroposterior length in the reported specimen (L2 in the table S2) is slightly shorter than in other fossil species such as *G. auaritae* and *G. goliath,* whereas it is bigger than in the rest of giant species (see table S2). On the other hand, the lateromedial widths (A3 and A4 in the table S2) are narrower than in *G. goliath* and bigger than in the rest of giant species (see table S2).

The parietal foramen is anteriorly directed like in *G. goliath, G. stehlini* and *G. simonyi* and unlike in *G. intermedia* and *G. bravoana,* where it is directed ventrally. The anterior surface between the lateral parietal cranial crests is similar to that of *G. goliath*: it resembles a triangle and is narrow in comparison with the broad anterior surface of, for example, *G. galloti* [20.].

- Pterygoid

The teeth present in this bone in the specimen reported herein are distributed in two branches forming a V-shape, being the medial branch (8 teeth) slightly longer than the lateral one (5 teeth), in a similar way to *G. simonyi* and the referred material of *G. auaritae* and *G. goliath*. The distribution of these teeth in adult individuals of *Gallotia* vary between a unique branch in *G. galloti, G. caesaris, G. atlantica* and *G. bravoana,* a patch on a raised bony prominence in *G. stehlini,* and an asymmetric V-shaped distribution in *G. intermedia, G. goliath, G. auaritae* and *G. simonyi* [8, 20] (see table S3).

- Dentary

The dentaries of the reported specimen are slightly shorter than other fossils of giant species such as the holotype of *G. auaritae* or *G. goliath*, they are a third longer than *G. stehlini* and *G. simonyi*, and are the twice as long as *G. bravoana* and *G. intermedia* (see table S3). The posterodorsal process apparently finishes at the level of the anterior half of the dorsal process of the coronoid, in a similar way to *G. bravoana, G. simonyi* and *G. stehlini* and unlike to *G. goliath* where it finishes at the level of the posterior part of the dorsal process of the coronoid. The anterior extension of the surangular, separating the posterodorsal and posteroventral processes of the dentary, is pointed and apparently finishes at the level of the coronoid like in the others giant taxa. The subdental shelf, measured between the most proximal end of the dentary and the last tooth of the dentary, has a similar length to the holotype of *G. auaritae*, is shorter than *G. goliath* and longer than in *G. bravoana, G. simonyi, G. stehlini* and *G. intermedia* (table S3). Lastly, the number of teeth positions in the reported specimen is lower than in *G. goliath* and the holotype of *G. auaritae*, and higher than in *G. stehlini, G. simonyi, G. intermedia* and *G. bravoana* [8, 44-45] (see table S3).

- Coronoid

In lateral view, the anterolateral process is long and has a rectangular shape like in *G. stehlini* and unlike in *G. goliath, G. bravoana, G. intermedia* and *G. simonyi* where it is short and triangular. In medial view, the anteromedial process is triangular like in *G. bravoana, G. goliath, G. intermedia* and *G. simonyi* and unlike the narrow and acuminate shape of *G. stehlini.* This process anteriorly surpasses the last tooth as occurs in *G. goliath* or *G. intermedia.*

- Dentition

The distribution of the monocuspid or bicuspid teeth in anterior position and tricuspid teeth in posterior position in the reported specimen is similar to that of *G. galloti*, *G. atlantica*, *G. caesaris*, *G. intermedia*, *G. simonyi* and that of the holotype of *G. auaritae* [5, 8, 20]. In contrast, in *G. stehlini* most teeth are multicuspid, whereas in *G. goliath* they are mainly bicuspid, with some tricuspid, and being able to reach four cusps [6].

Fossil record and distribution of extant species

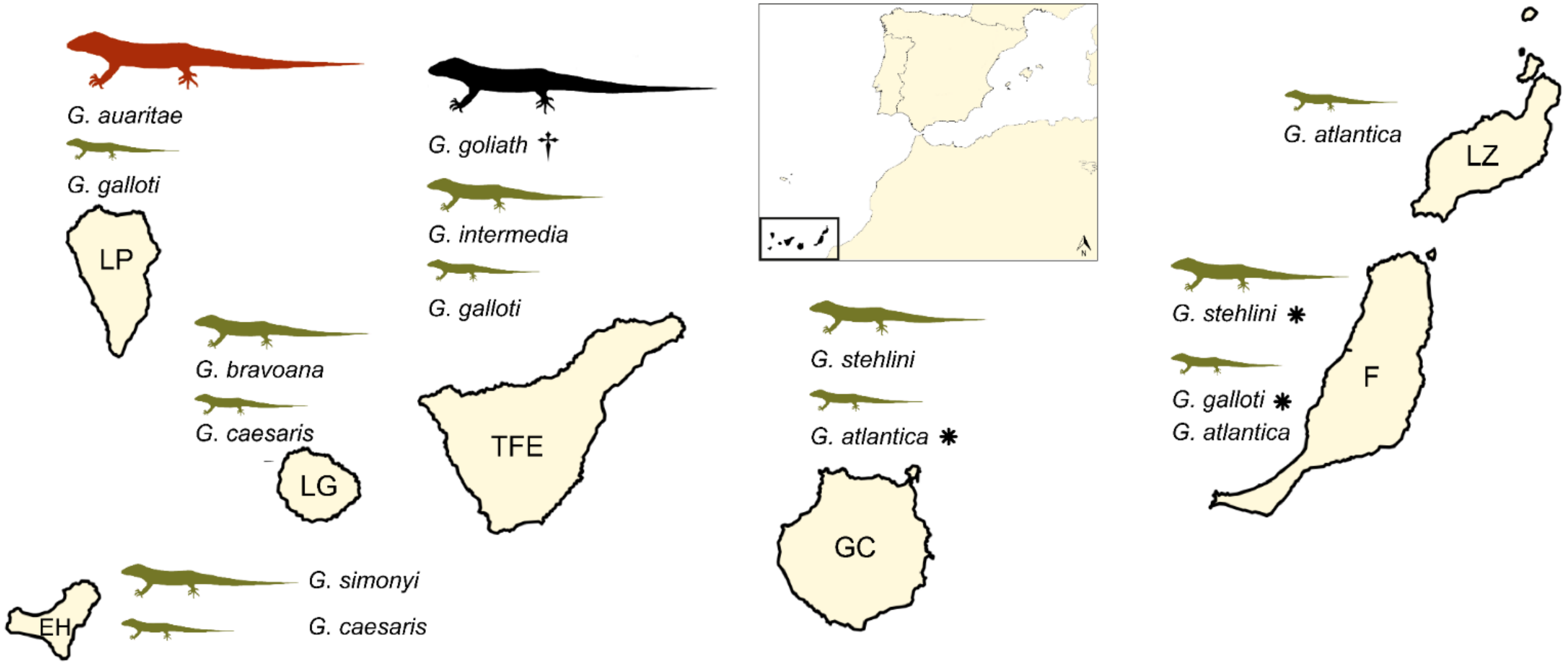

Figure S3. Distribution of the fossil and extant species of the genus *Gallotia*. Black shape, extinct species; Red shape, studied species (*G. auaritae*); Green shape, fossil and extant species; Asterisk, introduced species.

Table S1. Fossil record of the genus *Gallotia* in the Canary Islands. * Introduced species.

Taxa	Synonymous	Island	Fossil/Extant	Site	Age
Gallotia auaritae	*Lacerta simonyi, L. goliath, G. simonyi, G. simonyi auaritae*	La Palma	Yes/?	Roque de Mazo (locality type)	Holocene
				Los Llanos de Aridane	Holocene
				Cueva Los Murciélagos, Los Tilos	Upper Pleistocene
				La Puntilla	
Gallotia goliath	*L. goliath, L. máxima, G. máxima, G. simonyi*	Tenerife	Yes/No	Acantilado de Martiánez, Puerto	Early Pleistocene

			de la Cruz (locality type)<br>Costa de El Draguillo<br>San Andrés<br>Malpaís de Güimar<br>La Orotava<br>Santa Úrsula<br>Punta del Hidalgo<br>Punta de Teno<br>Barranco las Moraditas

Gallotia bravoana	*G. simonyi, G. simonyi gomerana, G. goliath bravoana*	La Gomera	Yes/Yes	Barranco de Chinguarime (locality type)<br>Barranco de Machal<br>Barranco de Santiago<br>Agulo	Early Pleistocene
Gallotia stehlini	*L. stehlini*	Gran Canaria, Fuerteventura*	Yes/Yes	Ingenio (Gran Canaria)<br>La Aldea de San Nicolás (Gran Canaria)<br>La Isleta (Gran Canaria)	Pleistocene

Gallotia simonyi	*L. simonyi, G. simonyi machadoi*	El Hierro	Yes/Yes	Playa de La Arena Guinea (locality type)<br>Cuaclo de Las Moleras (La Dehesa)<br>El Julan	Holocene
Gallotia intermedia	*G. simonyi*	Tenerife	Yes/Yes	Risco de La Jaqueta in the Acantilado de Los Gigantes (Tenerife, locality type)	Holocene

Gallotia caesaris	*L. caesaris, G. galloti gomerae*	La Gomera, El Hierro	Yes/Yes	Barranco de Chinguarime (La Gomera)<br>Barranco de Machal (L Gomera)	Early Pleistocene
Gallotia galloti	*L. galloti*	Tenerife, La Palma, Fuerteventura*	Yes/Yes	Costa de El Draguillo (Tenerife)<br>Acantilado de Martiánez, Puerto de la Cruz (Tenerife)	Early Pleistocene<br>Pleistocene
Gallotia atlantica		Lanzarote, Fuerteventura, Gran Canaria*			

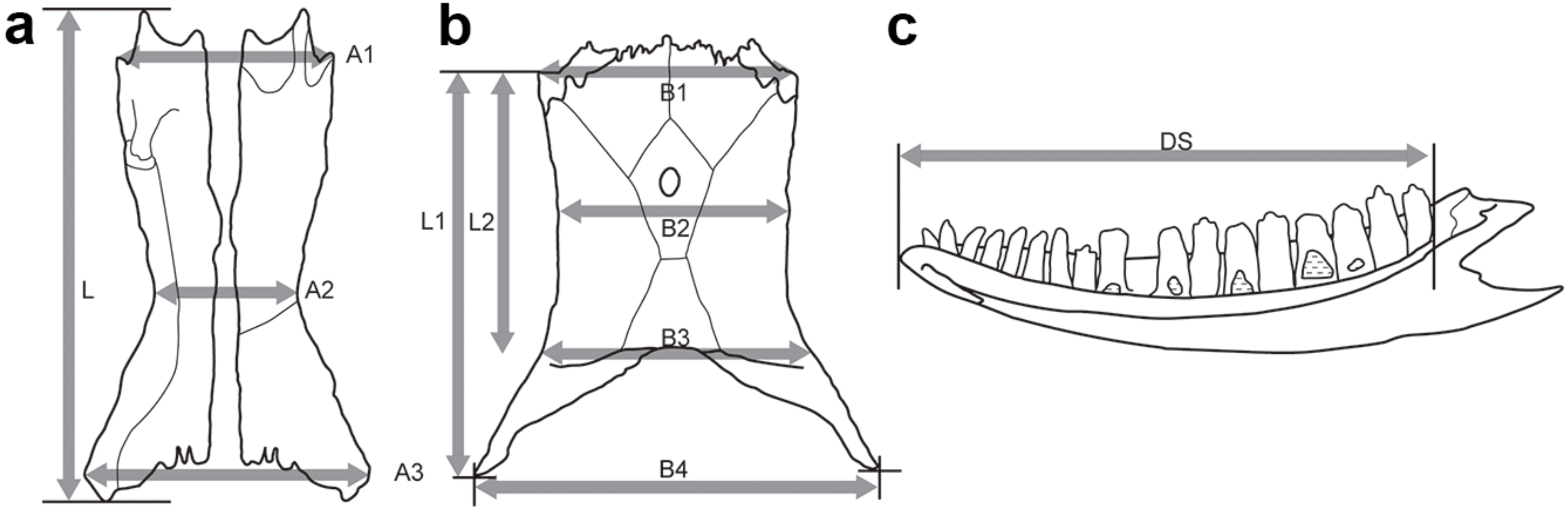

Figure S4. Schematic drawings of frontal, parietal and right dentary in ventral (A), dorsal (B-C) and lateral (D) views, showing the measurements taken for tables S1 and S2. A1, anterior mediolaterally wide of the frontal; A2, half mediolaterally wide of the frontal; A3, posterior mediolaterally wide of the frontal; B1, anterior mediolaterally wide of the parietal; A2, half mediolaterally wide of the parietal; A3, posterior mediolaterally wide of the parietal; DS, subdental shelf length; L, length of the frontal; L1-2, length of parietal.

Table S2. Measurements of the frontal and the parietal of the giant fossil and extant lizard of the genus *Gallotia*.

**Taxa**	**FRONTAL**				**PARIETAL**						
**Mm**	**Length**	**A1**	**A2**	**A3**	**L1**	**L2**	**L3**	**A1**	**A2**	**A3**	**A4**
PCCRULL1169	37.7	14.3	13.2	18.9	X	26	7.2	16.7	19	16.5*	X
Gallotia auaritae	47.1*	15.9*	15.5	25.9*	37.7*	31.5	27.5	X	X	27*	32.9*
Gallotia goliath	42.5	18.2	16.9	27.3	X	32.3	23	26.6	22.7	30.9	X
Gallotia bravoana (Male, GB01/2015)	11.4	5.2	4.3	7.5	16.8	17.4	5.4	5.9	7.2	8	12.3
Gallotia bravoana (Female, GB02/2015)	12.6	6.7	5.8	7.4	18.5	16.5	6.7	8	8.4	11	15.6
Gallotia stehlini (TFMV-VT96)	20.9	4.5	7.5	10.9	26.5	20	9.8	12.6	11.9	16.6	21.4
Gallotia simonyi (GS-1/2015)	24.7	9.9	6.8	7.7	24.5	17	9.7	13.1	13.4	13.5	22.4

Gallotia intermedia (DZUL-2208)	14.7	7.5	6.4	8.4	16.6	12.9	8.8	10.1	10.1	11.4	17.8

*incomplete

Table S3. Measurements of the dentary, the maxillar and the pterygoid of the giant fossil and extant lizard of the genus *Gallotia*. *incomplete.

**Taxa**	**Dentary**				**Maxillary**		**Pterygoid**	
**mm**	**Mandible length**	**Dentary length**	**Subdental shelf length**	**Number teeth**	**Maxillary length**	**Number teeth**	**Distribution**	**Number teeth**
PCCRULL1169	69.1*	48.4*	44.4	27	44.3	23	V	13
Gallotia auaritae	53.2*	53.2*	43.8*	32-33	X	X	V	4-27
Gallotia goliath	119	67.5	57.2	32	X	X	X	X
				33-34			V	18
Gallotia bravoana (Male, GB01/2015)	31.1	17.4	14.3	19	16.2	17	Unique branch	7

Gallotia bravoana (Female, GB02/2015)	38.1	19.8	18.1	21	18.2	16	Unique branch	X
Gallotia stehlini (TFMV-VT96)	58.9	30.8	26.1	26	31.3	20	V	11
				26			Patch	0-25
Gallotia simonyi (GS-1/2015)	60.5	33	28.1	23	30.4	21	Unique branch	7
				21-26			V	4-27
Gallotia intermedia (DZUL-2208)	42	23.7	19.7	25	21.6	22	V	16

Supplementary Data S2. Microfocus X-ray computed tomography (microCT)

The specimens were analysed by microfocus X-ray computed tomography (microCT) at the Multidisciplinary Laboratory of the "Abdus Salam" International Centre of Theoretical Physics (Trieste, Italy), using a system specifically designed for the study of archaeological and paleontological materials (Tuniz et al. 2013).

The microCT acquisitions of the specimens were carried out by using a sealed X-ray source (Hamamatsu L8121-03) at a voltage of 110 kV, a current of 90 µA and with a focal spot size of 5 µm. The X-ray beam was filtered by a 1 mm-thick aluminium absorber. A set of 1440 projections of the samples were recorded over a total scan angle of 360° by a flat panel detector (Hamamatsu C7942SK-25). The resulting microCT slices were reconstructed using the commercial software DigiXCT (DIGISENS) in 32-bit format and obtaining an isotropic voxel size from about 20 to 40 µm (see table S3).

Table S4. microCT scan parameters used for *Galliota* specimens. Al: aluminum.

TAXON	CATALOGUE NO.	DEPOSIT	SEX AND ONTOGENY STAGE	KV VOLTAGE	µA CURRENT	FILTER	PROJECTIONS	VOXEL SIZE (MICRON)
G. auaritae	PCCRULL1169	DBAEG, ULL	Adult	110	90	1 mm Al	1440	44.88
G. goliath	H010	DBAEG, ULL	Adult	110	90	1 mm Al	1440	44.88
G. atlantica	TFMC-VT 120	MUNA	Male adult	110	90	1 mm Al	1440	21.42
G. bravoana	Gb-1/2015	UMCG	Male adult	110	90	1 mm Al	1440	39.71
G. bravoana	Gb-2/2015	UMCG	Female adult	110	90	1 mm Al	1440	39.71
G. caesaris caesaris	Gc-1/2015	UMCG	Male adult	110	90	1 mm Al	1440	39.71
G. caesaris caesaris	Gc-2/2015	UMCG	Female adult	110	90	1 mm Al	1440	39.71
G. caesaris gomerae	TFMC-VT 28	MUNA	Male adult	110	90	1 mm Al	1440	21.42

G. galloti galloti	TFMV-VT 115	MUNA	Male adult	110	90	1 mm Al	1440	39.71
G. galloti palmae	TFMV-VT 22	MUNA	Adult	110	90	1 mm Al	1440	39.71
G. intermedia	DZUL-2208	DBAEG, ULL	Male adult	110	90	1 mm Al	1440	39.71
G. simonyi	Gs-1/2015	UMAH	Male adult	110	90	1 mm Al	1440	39.71
G. simonyi	Gs-2/2015	UMAH	Female adult	110	90	1 mm Al	1440	39.71
G. stehlini	TFMV-VT 96	MUNA	Male	110	90	1 mm Al	1440	39.71

Supplementary Data S3. Additional phylogenetic analysis

An additional phylogenetic analysis (with file S3a as the data matrix), including the poorly known *Gallotia goliath* was performed. The same settings as for the main analysis were used. The analysis yielded 17 MPTs (140.77 steps). The resulting general topology is similar to that of the main analysis, except that in the strict consensus tree (Supplementary Data S7) the relationships within the "*simonyi* group" are unresolved. This is related to the fact that the added taxon, *G. goliath*, is scored for very few characters. Note, however, that both the Adams consensus and the Majority Rule (Supplementary Data S7) consensus trees recover as sisters the fossil and *G. auaritae* (82% of the MPT's trees recover this topology). **Supplementary Data S3a (Nexus file of the data matrix), S3b (Nexus file of the data matrix, with *Gallotia goliath* included), and Supplementary Data S4 and S6, the lists of apomorphies for the main analysis and for the analysis including *Gallotia goliath*, respectively, are all provided as separate files.**

S5. Three most parsimonious trees resulting from the main analysis

1

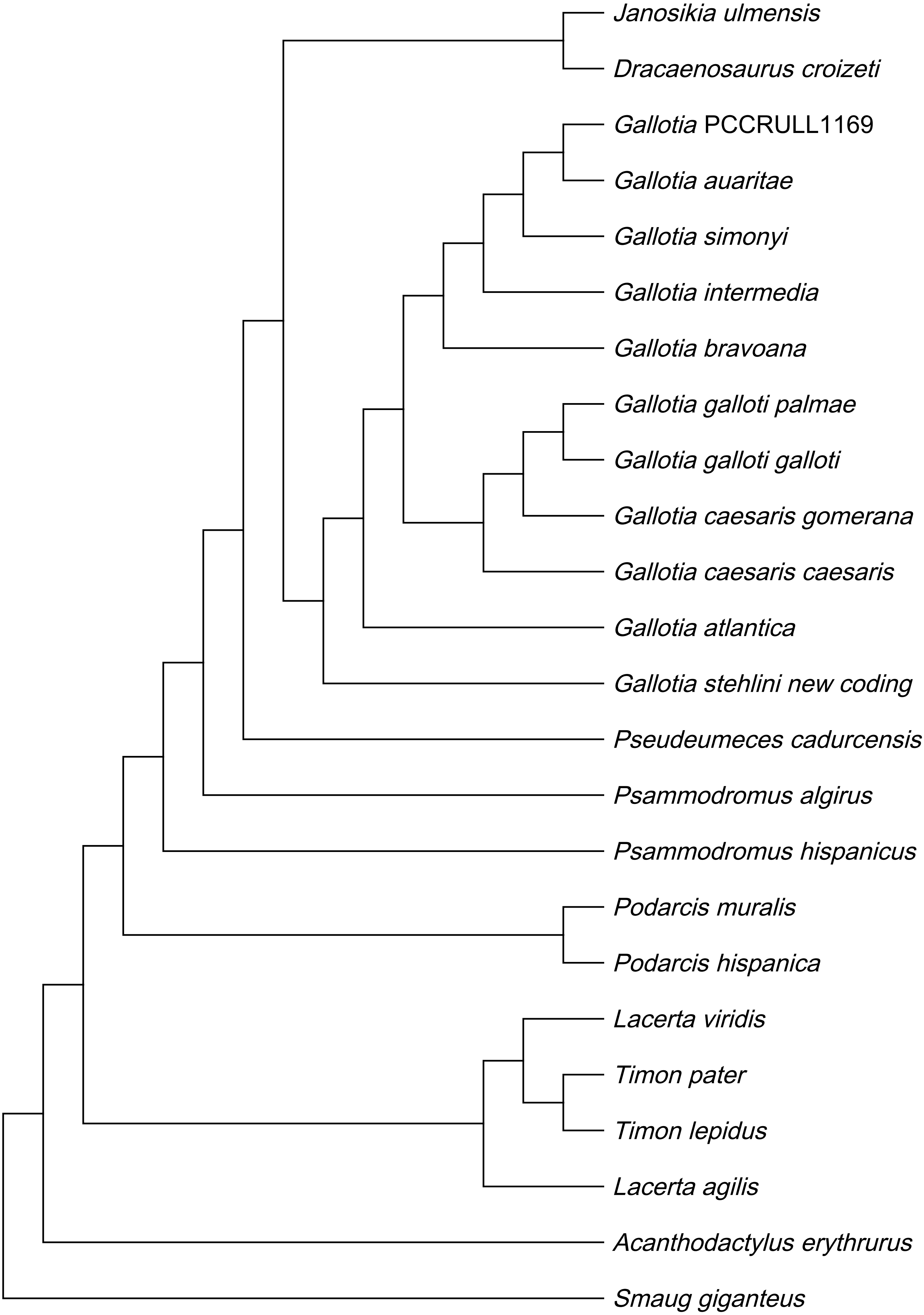

2

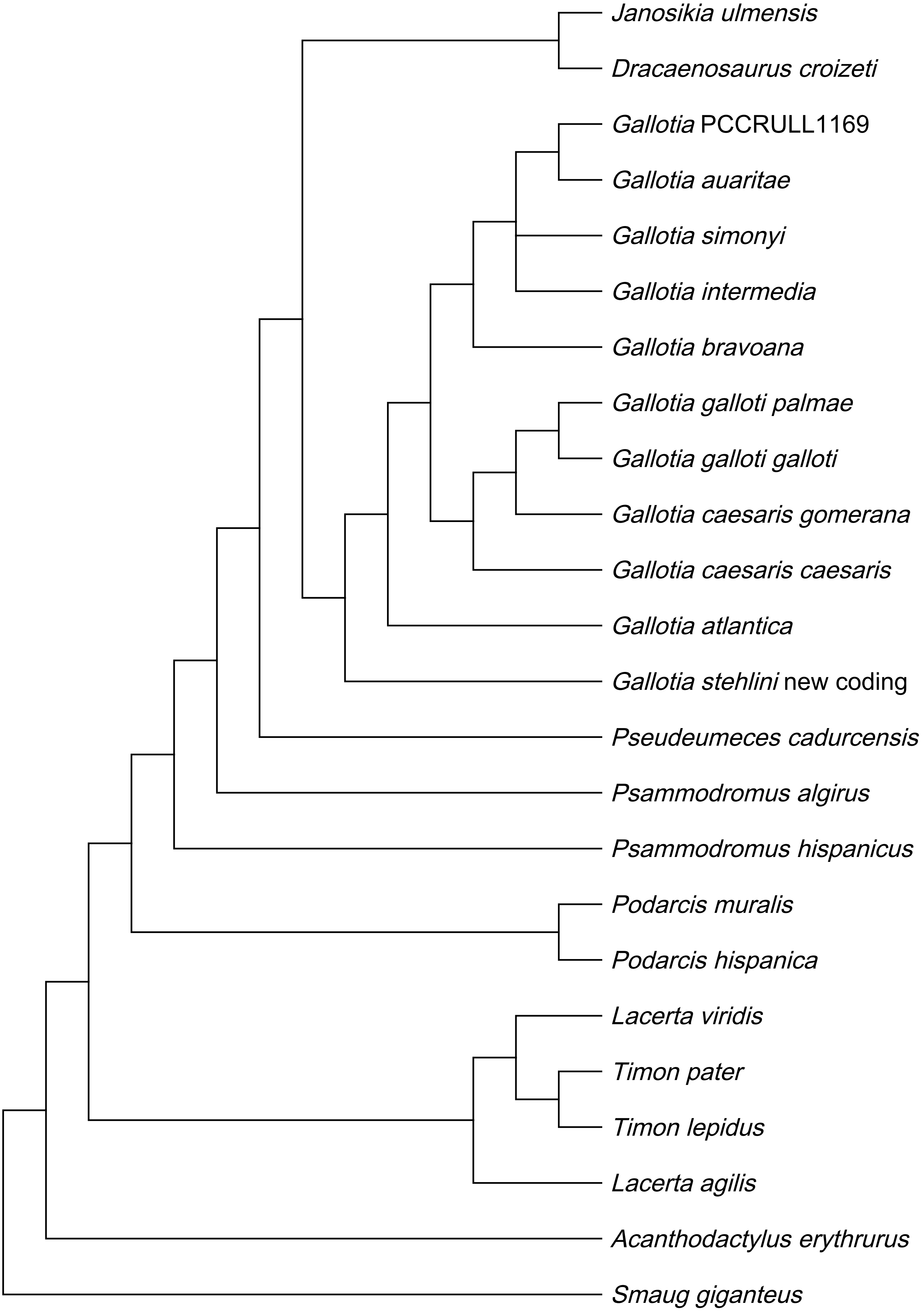
Janosikia ulmensis
Dracaenosaurus croizeti
Gallotia PCCRULL1169
Gallotia auaritae
Gallotia simonyi
Gallotia intermedia
Gallotia bravoana
Gallotia galloti palmae
Gallotia galloti galloti
Gallotia caesaris gomerana
Gallotia caesaris caesaris
Gallotia atlantica
Gallotia stehlini new coding
Pseudeumeces cadurcensis
Psammodromus algirus
Psammodromus hispanicus
Podarcis muralis
Podarcis hispanica
Lacerta viridis
Timon pater
Timon lepidus
Lacerta agilis
Acanthodactylus erythrurus
Smaug giganteus

3

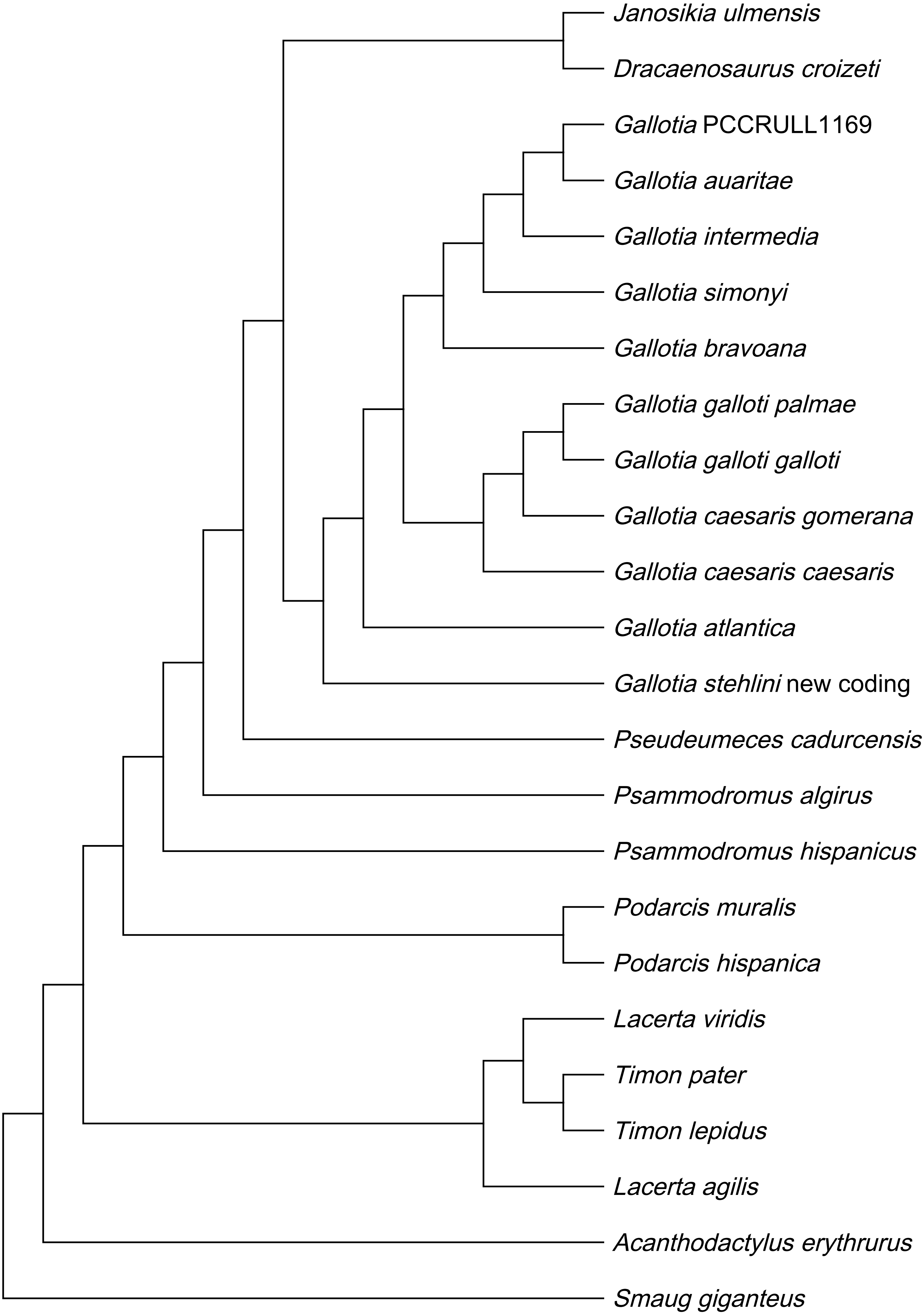
Janosikia ulmensis
Dracaenosaurus croizeti
Gallotia PCCRULL1169
Gallotia auaritae
Gallotia intermedia
Gallotia simonyi
Gallotia bravoana
Gallotia galloti palmae
Gallotia galloti galloti
Gallotia caesaris gomerana
Gallotia caesaris caesaris
Gallotia atlantica
Gallotia stehlini new coding
Pseudeumeces cadurcensis
Psammodromus algirus
Psammodromus hispanicus
Podarcis muralis
Podarcis hispanica
Lacerta viridis
Timon pater
Timon lepidus
Lacerta agilis
Acanthodactylus erythrurus
Smaug giganteus

S7. Strict, Majority Rule and Adams consensus trees for the analysis including *Gallotia goliath.*

Strict consensus tree

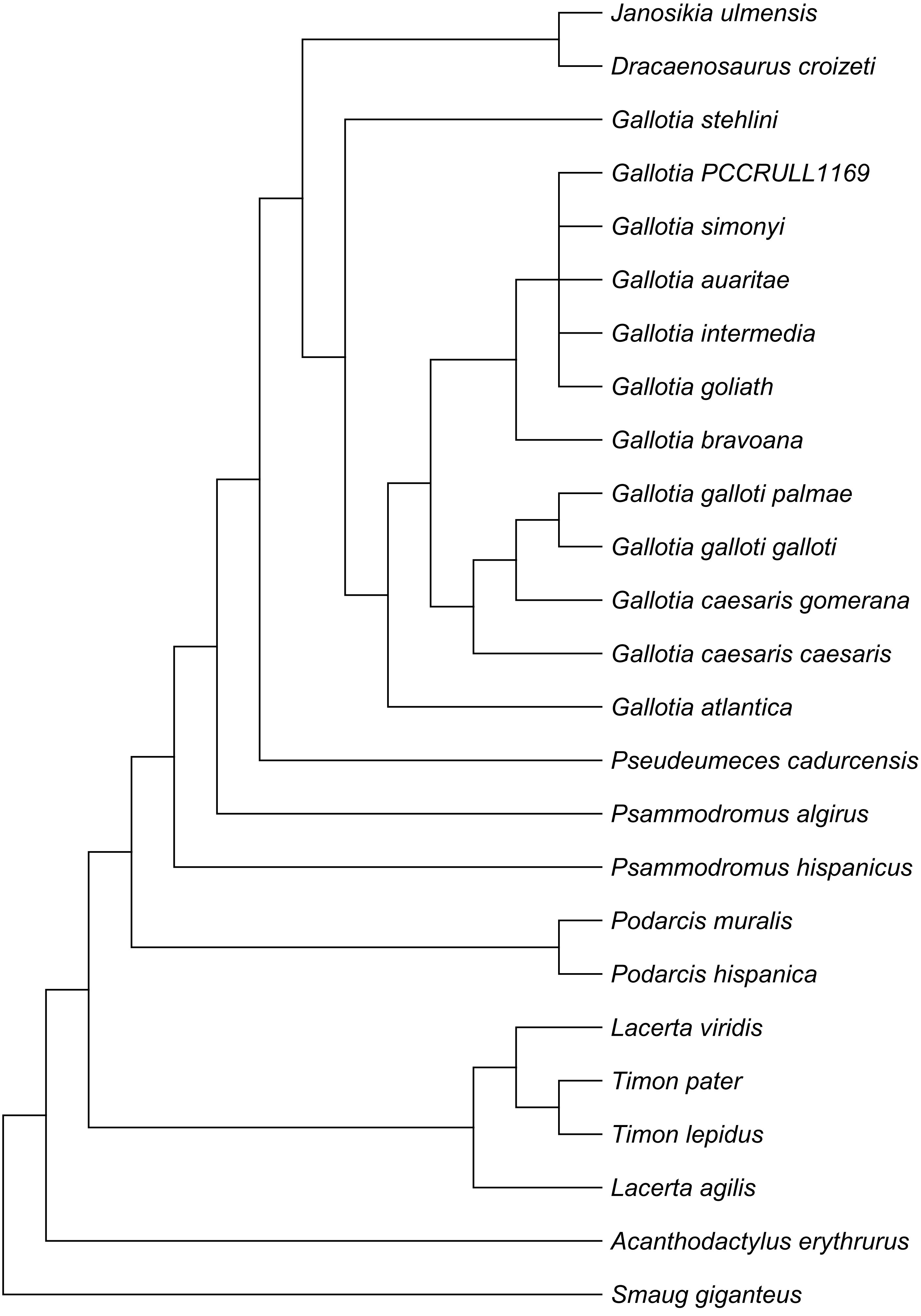

Majority-rule consensus tree

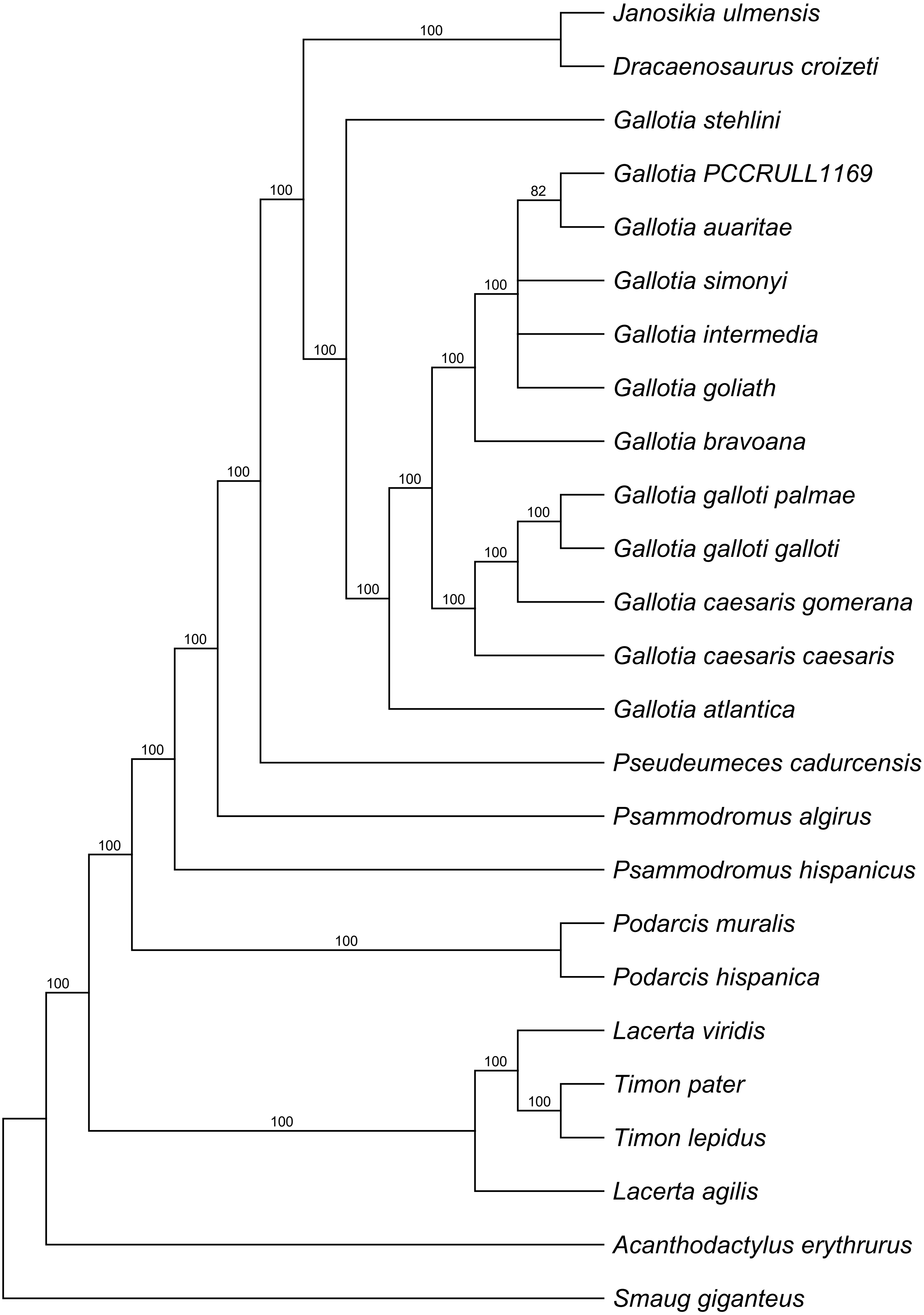

Adams consensus tree

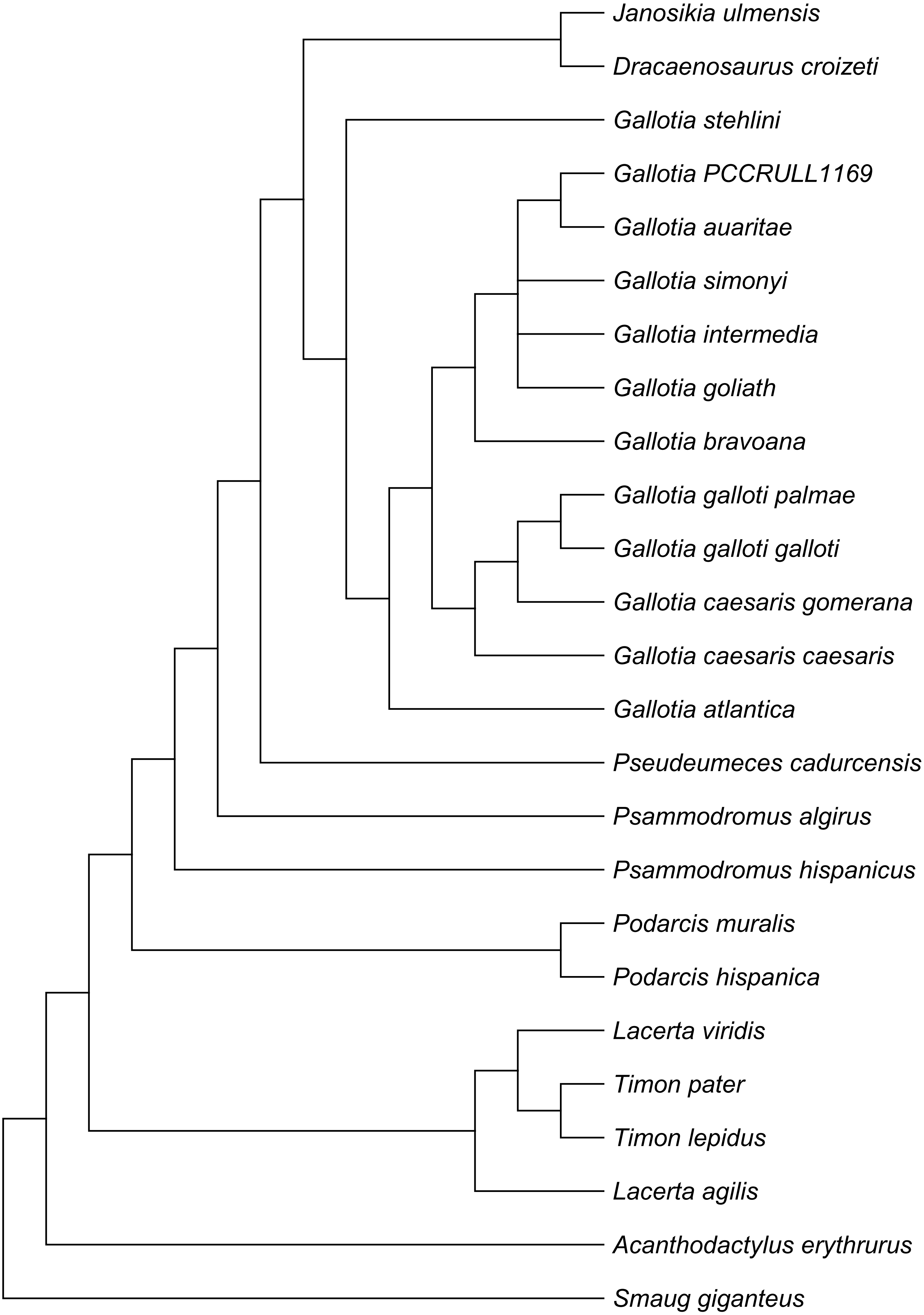

References

37. López-Jurado, L.F. Los reptiles fósiles de la Isla de Gran Canaria (Islas Canarias). *Bonner Zoologische Beiträge* **36**: 355-364. (1985).

38. Bolet, A., Fortuny, J. *Gallotia stehlini* (Lacertidae) from the Pleistocene of Gran Canaria: new data from microCt-scans of unpublished specimens. In: *XIV Annual Meeting of the European Association of Vertebrate Palaeontologists* 6-10 July 2016, Haarlem, The Netherlands. P. 73. (2016)

39. Carracedo, J. C., Day, S. J., Guillou, H. & Pérez Torrado, F. J. Giant Quaternary landslides in the evolution of La Palma and El Hierro, Canary Islands. *J. Volcanol. Geotherm. Res.* **94**, 169-190. (1999b)

40. Colmenero, J. R, De La Nuez, J., Casillas, R. & Castillo, C. Epiclastic deposits associated with large-scale landslides and the formation of erosive calderas in oceanic islands: The example of the La Palma Island (Canary Archipelago). *Geomorphology* **177-178**: 108-127. DOI:10.1016/j.geomorph.2012.07.019. (2012)

41. Vegas, J., Channing, A., Anderson, C. L., Pais, J., Santos, A. & Hernández, M. V. of referencing in Los fósiles vegetales de la Caldera de Taburiente: investigación, geoconservación y divulgación del patrimonio paleontológico en la isla de La Palma. (eds. J. Vegas, A. Salazar, E. Díaz-Martínez & C. Marchán). Patrimonio geológico, un recurso para el desarrollo. Cuadernos del Museo Geominero, nº 15. Instituto Geológico y Minero de España, (Madrid, 2013).

42. García-Romero, M. E., Vegas, J., Marfil, R. & Baldonedo, J. L. Clay minerals as alteration products in basaltic volcaniclastic deposits of La Palma (Canary Islands, Spain). *Sediment. Geol.*, **174**, 237-253. (2005)

43. Alvarez-Ramis, M. C., Laamarti, N. & Vegas, J. A preliminary palynological study of epiclastic deposits from “Caldera de Taburiente”. La Palma island. Canary archipelago, Spain. *Plant Cell Biology and Development*, **11**, 50-57. (2000)

44. Izquierdo, I. Medina, A. L. & Hernández, J. J. Bones of giant lacertids from a new site on El Hierro (Canary Islands). *Amphibia-Reptilia*, **10(1),** 63-69. (1989).

45. Mateo, J. A. Lagarto gigante de La Palma – *Gallotia auaritae*. of referencing in Enciclopedia Virtual de los Vertebrados Españoles. (eds. Carrascal, L. M., Salvador, A.). (2009).
